# Single-cell morphometrics reveals ancestral principles of notochord development

**DOI:** 10.1242/dev.199430

**Published:** 2021-08-19

**Authors:** Toby G. R. Andrews, Wolfram Pönisch, Ewa K. Paluch, Benjamin J. Steventon, Elia Benito-Gutierrez

**Affiliations:** 1 Department of Zoology, University of Cambridge, Cambridge CB2 3EJ, UK; 2 Department of Physiology, Development and Neuroscience, University of Cambridge, Cambridge CB2 3EL, UK; 3 Department of Genetics, University of Cambridge, Cambridge CB2 3EH, UK

**Keywords:** Morphometrics, Single-cell, Amphioxus, Notochord, Morphogenesis, Evolution

## Abstract

Embryonic tissues are shaped by the dynamic behaviours of their constituent cells. To understand such cell behaviours and how they evolved, new approaches are needed to map out morphogenesis across different organisms. Here, we apply a quantitative approach to learn how the notochord forms during the development of amphioxus: a basally branching chordate. Using a single-cell morphometrics pipeline, we quantify the geometries of thousands of amphioxus notochord cells, and project them into a common mathematical space, termed morphospace. In morphospace, notochord cells disperse into branching trajectories of cell shape change, revealing a dynamic interplay between cell shape change and growth that collectively contributes to tissue elongation. By spatially mapping these trajectories, we identify conspicuous regional variation, both in developmental timing and trajectory topology. Finally, we show experimentally that, unlike ascidians but like vertebrates, posterior cell division is required in amphioxus to generate full notochord length, thereby suggesting this might be an ancestral chordate trait that is secondarily lost in ascidians. Altogether, our novel approach reveals that an unexpectedly complex scheme of notochord morphogenesis might have been present in the first chordates.

This article has an associated ‘The people behind the papers’ interview.

## Abstract (precis)

**Summary:** Single-cell morphometrics reveals that notochord development in amphioxus, a basally branching chordate, is driven by a complex set of cellular behaviours characterised by specific trajectories of cell shape change.

## INTRODUCTION

A major challenge in biology is to understand how individual cells coordinate their behaviours during embryogenesis to generate tissues of the correct geometry and size, and how these behaviours are modified through evolution to generate morphological novelty. The notochord is a pivotal case study in this context. It is a defining feature of the chordate body plan with diverse contributions to axial development. The notochord also has a simple geometry, as an elongate rod of mesodermal tissue occupying the embryonic axial midline (Stemple, 2005). During its development, the notochord exerts essential roles in body plan formation. This includes contributions to axis elongation and mechanical stabilisation of the body axis (Stemple, 2005; Segade et al., 2016; Xiong et al., 2018 preprint), and, in vertebrates, the secretion of organising signals that provide dorsoventral patterning information to the adjacent neural tube and somites (Placzek et al., 1991; Pourquié et al., 1993; Yamada et al., 1993). Although notochord development has been well characterised in olfactores (ascidians and vertebrates), the cell behaviours responsible for its formation in cephalochordates, the most basally branching chordate subphylum (Delsuc et al., 2006), remain unknown. The cephalochordate amphioxus provides a unique opportunity to fill this gap and further our understanding of morphogenetic principles common to all chordates, which are likely to have evolved from the common chordate ancestor.

In vertebrates and ascidians, the notochord develops from a broad field of mesodermal progenitors (termed chordamesoderm) that organise into an elongated rod of tissue by changing their shapes and spatial organisation. Notochord cells actively change their shape, and crawl between adjacent neighbours to intercalate and generate a single-file row. As a result, neighbouring cells are forced apart along the anteroposterior (AP) axis, resulting in tissue extension (Glickman et al., 2003; Munro and Odell, 2002; Shih and Keller, 1992). This process, in which tissue length is established at the expense of width, is termed convergent extension (Keller et al., 2000). To variable degrees in each species studied, convergent extension synergises with cell growth and proliferation to define total notochord length. This ranges from ascidians, where notochord morphogenesis occurs in a population of exactly 40 post-mitotic cells (Miyamoto, 1985; Veeman and Smith, 2013), to amniotes that extensively elongate the notochord primordium after gastrulation through proliferation of posterior axial progenitors, and widespread intercalatory cell division (Cambray and Wilson, 2002; Catala et al., 1996; Selleck and Stern, 1991). Cell intercalation is generally followed by vacuolation of individual notochord cells, which expands their volume and increases notochord length and rigidity (Adams et al., 1990; Bagnat and Gray, 2020; Bancroft and Bellairs, 1976; Ellis et al., 2013).

In amphioxus, the chordamesoderm is specified at the dorsal midline of the archenteron, the primitive gut cavity formed during gastrulation (Zhang et al., 1997). After gastrulation, the chordamesoderm evaginates, and cells interdigitate across the midline (Fig. S1). Eventually, the notochord establishes a trilaminar arrangement along the dorsoventral (DV) axis, including a central row of flattened cells in a ‘stack-of-coins’ pattern, flanked dorsally and ventrally by single-file rows of Müller cells (Conklin, 1932). The central ‘stack-of-coins’ is a feature shared with other chordates. In contrast, the Müller cells, the function of which is currently unknown, are unique to amphioxus (Bočina and Saraga-Babić, 2006; Holland and Holland, 1990; Flood, 1975). During amphioxus development, little proliferation has been reported in the notochord, except for cells at its posterior tip (Holland and Holland, 2006). Cell growth has also been described, and attributed to vacuolation (Hatschek, 1893). However, beyond these studies, a detailed understanding of the cellular behaviours responsible for formation and elongation of the amphioxus notochord remains lacking (Annona et al., 2015). This is in part due to the challenge of imaging amphioxus embryos *in vivo*. Although their small size and optical transparency make them an ideal system for high-resolution imaging, their rotational movement during embryogenesis impedes live imaging. This means other methods are needed to infer cell behaviour.

To build a complete picture of cell shape changes during amphioxus notochord development, we present a pipeline to segment and quantify the shapes of thousands of individual notochord cells, and embed them in a single-cell morphospace; a mathematical space in which cells disperse into trajectories of shape change. This morphospace reveals branching trajectories of cell shape change that remodel a common progenitor morphology towards a diversity of forms during notochord elongation. Focussing on a specific region, we use geometric modelling to determine how specific geometric transformations contribute to global cell shape transitions. This analysis identifies a synergistic relationship between cell shape change and growth, that adds axial length to the notochord. By spatially mapping cells to their position along the AP axis, we show that cells in different positions along the AP axis progress towards common morphologies both at different times, and through different morphogenetic paths, hinting at regionalisation of cell behaviour across the AP axis. Finally, we use cell labelling and functional perturbations to test the role of cell division in notochord elongation, and identify a requirement for posterior progenitors in providing the cell number required for full tissue elongation. In summary, our approach reveals the amphioxus notochord to be morphologically more complex and heterogenous than previously thought. Adding to existing knowledge on vertebrate and ascidian notogenesis, our work reveals a highly conserved repertoire of morphogenetic processes throughout chordates, suggesting these might have a common evolutionary origin in the first chordates.

## RESULTS

### A single-cell morphospace captures branching trajectories of shape differentiation specific to cell type

**Fig. 1.**
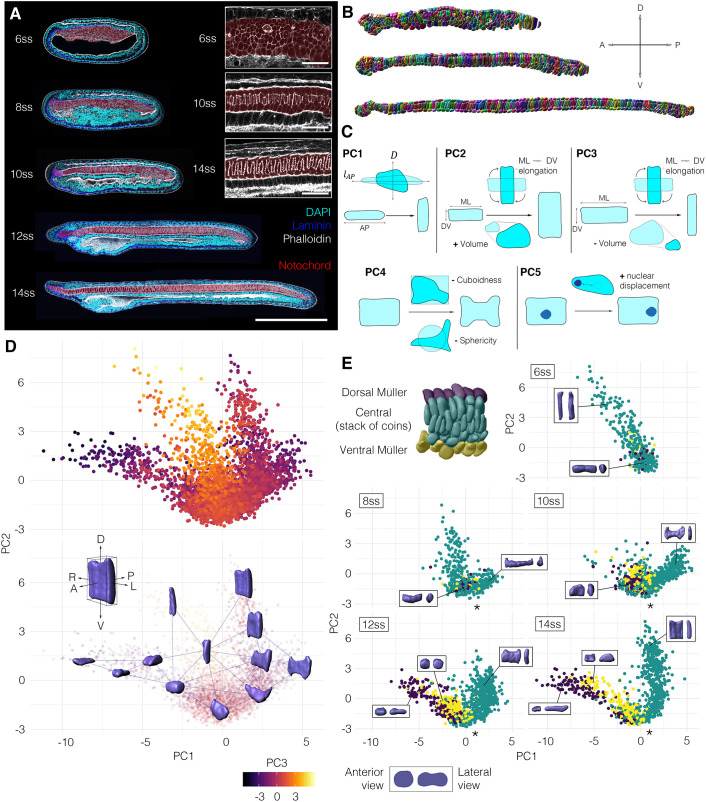
**A single-cell morphospace captures notochord cell shape diversity.** (A-E) Steps 1-4 of the morphospatial embedding pipeline (see Materials and Methods). (A) Amphioxus embryos at successive somite stages stained with phalloidin to mark cortical actin, which was used for manual cell segmentation. Embryos are also immunostained for laminin (extracellular matrix) and acetylated tubulin (cilia). Notochord is false-coloured in red. (B) Images of fully segmented notochords, from 8 ss (top), 10 ss (middle) and 12 ss (bottom). (C) Cell shape metrics correlated with the first five PCs (direction of correlation indicated by plus and minus). (D) Morphospace containing all notochord cells plotted against PC1 and PC2. (Top) Notochord morphospace for all cells and stages, colour code for PC3. (Bottom) Notochord morphospace with representative surfaces from cell segmentation data in anterolateral view. (E) Morphospace filtered by stage, colour-coded for DV position as indicated on a 8 ss notochord fragment (top left). Representative cells are shown in inlays, in anterior (left) and lateral (right) views. Asterisks mark bifurcation resolving central and Müller cells. All embryos and notochords are in lateral view, anterior side facing left. *n=*3796 cells, five stages, three embryos per stage.

In amphioxus, notochord formation involves a radical change in cellular organisation from a seemingly disorganised array of rounded cells at the 6-somite stage (ss), to a regular trilaminar array at 14 ss. This consists of central cuboidal cells in a stack-of-coins pattern sandwiched between two rows of Müller cells (Fig. 1A, Fig. S1) (Hatschek, 1893; Conklin, 1932). To reconstruct trajectories of single-cell shape change involved in this maturation process, we assembled a cell segmentation and morphospace embedding pipeline (Fig. S2). First, we built a dataset of manually segmented notochord cells at successive stages of elongation, using embryos stained with phalloidin to mark cortical actin (Fig. 1A,B). 3796 cells were segmented, from 15 notochords across five developmental stages. We next quantified a series of 15 geometrical parameters for each cell, relating to shape, size and orientation (Fig. 1C; see Materials and Methods). To identify the major axes of variation between cells, we then performed a principal component analysis (PCA) on these measurements (Fig. S3). In doing so, we found that 86.2% of cell shape variability was explained by five eigenvectors (Fig. S3A). PC1 (36.7% variation) describes relative changes in AP length and transverse cross-sectional area, which we define as AP anisotropy (Fig. S3D). PC2 (20.9% variation) describes correlated changes in the direction of cell elongation on the transverse plane and cell volume (Fig. S3E). In contrast, PC3 (14.1% variation) describes anti-correlated changes in transverse cell orientation and cell volume (Fig. S3F). Finally, PC4 (9.7% variation) describes surface convolution (Fig. S3G), and PC5 (4.8% variation) describes nuclear displacement from the centre of mass (Fig. S3H) (Fig. 1C). These components offered a framework to visualise geometric variation between notochord cells in a low-dimensional space.

We next plotted cells against the first three PCs to construct a developmental morphospace. In morphospace, cells organised in a continuous pattern, with three primary branches, and this was reproducible across all embryos in the dataset (Fig. 1D, Fig. S4). To determine how cells traverse morphospace during development, we subdivided the data by stage. At 6 ss, all cells occupied the central branch of morphospace, defined by tall DV-elongated morphologies (Fig. 1E, 6 ss). Over time, cells lost these morphologies and ‘flowed’ from the central branch into a bifurcation at the base of the plot (at the level of −1.5 on PC1), resolving two distinct trajectories of shape change (Fig. 1E, asterisk). By categorising cells based on relative DV position, we found the bifurcation to yield independent trajectories for the two main cell types in the notochord: the central cells and Müller cells (Fig. 1E, 8-14 ss). While the Müller cells became elongated along the AP axis at the expense of their transverse area (decreasing on PC1, anti-correlated with AP anisotropy), the central cells spread out on a transverse plane at the expense of their length (increasing on PC1). The trajectory followed by Müller cells was further divided in sub-trajectories for the dorsal and ventral rows, based on distinctive levels of AP anisotropy (Fig. 1E, yellow and blue points). Although cells from later stages populated more distant positions in morphospace with respect to the bifurcation, a continuum persisted at each stage between immature and differentiated states, hinting at temporal variation in shared cell shape transitions. Overall, morphospace embedding reveals a progressive diversification of cell morphology during notochord development, in which a common progenitor is remodelled to generate a diversity of cell morphologies.

### Single-cell morphometrics highlights two major shape transitions in central notochord cells

**Fig. 2.**
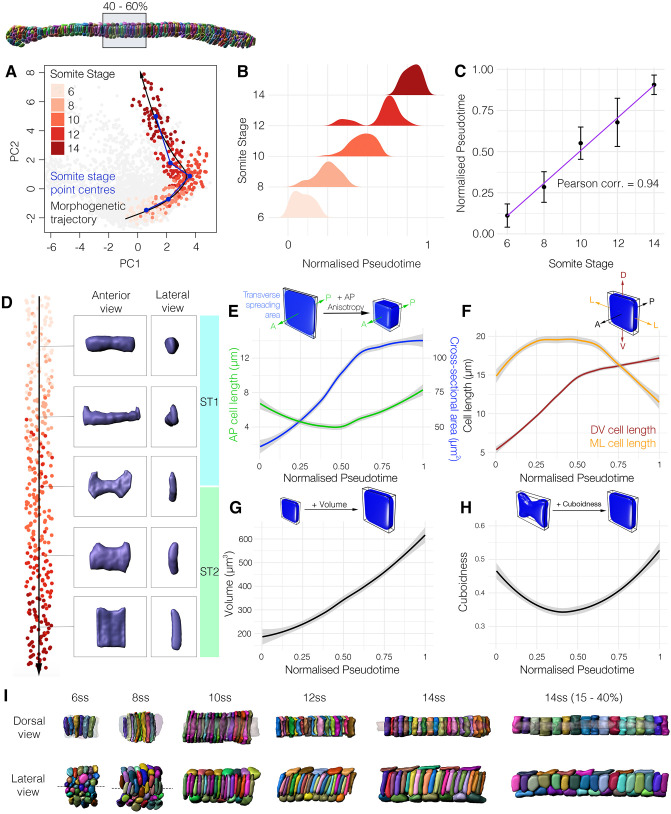
**The 40-60% central notochord cell shape trajectory.** (A) Notochord cells from the central layer at the 40-60% AP level projected into morphospace, coloured by stage. Blue segmented line connects point cloud centres for each stage; black curve shows the inferred trajectory calculated with Slingshot (Street et al., 2018). (B) Density plots showing frequency of cells across pseudotime for each somite stage. (C) Mean pseudotemporal distance for cells of each somite stage ±1 s.d with linear regression. (D) All 40-60% central layer notochord cells ordered across the inferred trajectory, with individual cells illustrated. (E-H) Changes in specific geometric parameters along the 40-60% central cell trajectory. (E) PC1 is anti-correlated with AP anisotropy, derived from transitions in cell AP length and cross-sectional area. (F) PC2 is positively correlated with the DV:ML aspect ratio, inferred from DV and ML bounding box lengths. (G) PC3 is anti-correlated with cell volume. (H) PC4 is negatively correlated with cuboidness. (I) Groups of segmented cells from the 40-60% level, shown in dorsal and lateral views at successive stages. In dorsal view, Müller cells are transparent. Dashed lines show DV position for dorsal views at 6 ss and 8 ss. *n=*344, 40-60% cells; *n*=360, 15-40% cells.

From this global view of shape differentiation, we next sought to refine a single shape trajectory for notochord cells by studying those in a specific region along the anteroposterior axis (Fig. 2A). Central layer cells from the trunk region (40-60% level of the AP axis) exhibited a continuous distribution across morphospace. This is illustrated by a partial overlap of the point clouds for cells of successive stages, suggesting that cells from each stage are heterogenous in their extent of shape differentiation (Fig. 2A). We next used trajectory inference to order these cells along a pseudotemporal axis, guided by prior information on temporal transitions from somite stages (Street et al., 2018). The inferred pseudotemporal axis passed through the point clouds for each somite stage in correct temporal sequence, and fitted closely to their mean PC coordinates (Fig. 2A, compare blue and black lines). By plotting the frequency of cells across pseudotime for each somite stage, we found cell shape transitions to be tightly controlled across developmental time, as measured by somite stage (Fig. 2B). Furthermore, we identified a strong positive correlation between pseudotime and somite stage (Pearson correlation=0.94) (Fig. 2C). This validated the inferred pseudotemporal axis as a vector for expressing the directionality and temporal progression of cell shape changes through morphospace.

We next asked how cell shape changes across the 40-60% central cell trajectory. We examined this qualitatively, by selecting cells from evenly spaced intervals across pseudotime (Fig. 2D, Movie 1), and quantitatively, by analysing changes in the major correlates of each PC (Fig. 2E-H). Together, our results highlight two major shape transitions that occur sequentially in central notochord cells.

In the first major shape transition (‘ST1’, 6-10 ss), cells transition from a mediolaterally (ML)-elongated morphology to a flattened morphology spread across the transverse plane, with flared margins similar in shape to a bowtie (Fig. 2D, ST1). In this process, cells decline in AP anisotropy (anti-correlated with PC1; Fig. 2E, Fig. S1B,S5F), accounted for by an increase in traverse cross-sectional area at the expense of AP length (Fig. 2E). In parallel, the transverse shape of each cell changes due to an increase in the DV:ML ratio (positively correlated with PC2, Fig. 2F, Fig. S5L). Cells elongate on both the ML and DV axes during ST1, but the earlier plateauing of ML length leads to anisotropic elongation along the DV axis (Fig. 2F). During ST1, we also found an increase in volume (anti-correlated with PC3, Fig. 2G, Fig. S3C), and a decline in cuboidness, which reflects how faithfully the cell fits its object-oriented bounding box (anti-correlated with PC4, Fig. 2H, Fig. S3G). This means that the total spreading area of the cell is increased beyond its cross-sectional area through surface convolution. Collectively, these geometric transitions describe formation of a bowtie morphology at 10 ss, which occupies a distinct domain in morphospace that we term the ‘bowtie domain’ (Fig. 2A,D).

During the second major shape transition (‘ST2’, 10-14 ss), cells lose the bowtie morphology, and acquire tall cuboidal shapes (Fig. 2D, ST2). Underlying these global changes, AP-oriented anisotropy now increases, due to an increase in AP length without further change in cross-sectional area (Fig. 2E, Fig. S4F). Meanwhile, the DV:ML ratio continues to increase due to a further slow increase in DV length, coupled to an almost 50% decrease in ML length (Fig. 2F, Fig. S5L). Volume undergoes a further net increase during ST2 (Fig. 2G). However, cuboidness and sphericity now increase, reflecting a decline in cell surface convolution (Fig. 2H, Fig. S5G,H). This corresponds to a lesser spread of the cell beyond its transverse cross-sectional area, and loss of the bowtie morphology (Fig. 2D). In summary, decomposition of the PCs exposed dynamic profiles of AP anisotropy, DV:ML ratio, growth and surface convolution as the primary geometric transformations underlying both major shape transitions that define the central cell trajectory.

To investigate how these major shape transitions in central notochord cells correspond to those in multicellular organisation, we segmented neighbourhoods of adjacent cells at the 40-60% level, and aligned changes in their organisation with the transitions defined in ST1 and ST2 (Fig. 2I). At 6 ss, all cells were elongated along their ML axes, touching both the left and right margins of the chordamesoderm (Fig. 2I, dorsal view, 6 ss). Because this stage precedes most of notochord AP elongation (Fig. 1A), ML intercalation cannot generate further tissue length. Instead, we noted an increase in the number of cell layers on the DV axis between 3 ss and 6 ss, suggesting that ML intercalation instead promotes convergent thickening (Fig. 2I, lateral view; Fig. S1B*,* compare 3ss and 6ss). During ST1 (6-10 ss), in which central notochord cells increase their transverse spread and adopt a bowtie morphology, we observed a process of intercalation oriented along the DV axis that reduces the number of cell layers from 6 to 3 (Fig. 2I, lateral view, 6-10 ss). During DV intercalation, cell neighbourhoods elongated on the AP axis, despite the AP shortening of individual cells (Fig. 2E,I). DV intercalation was followed by a later phase of neighbourhood elongation in the absence of further cell rearrangement, coinciding with ST2, in which cells increased in both AP length and volume (Fig. 2E,G,I 10-14 ss). This second elongation phase was more pronounced in the pharyngeal region (Fig. 2I, compare 14 ss 40-60% and 25-40%). In summary, the sequential shape changes of the central cell trajectory align with distinct phases of notochord elongation, involving, first, DV cell intercalation and, second, AP cell elongation.

### Geometric modelling reveals a requirement for growth in coupling convergence and extension

**Fig. 3.**
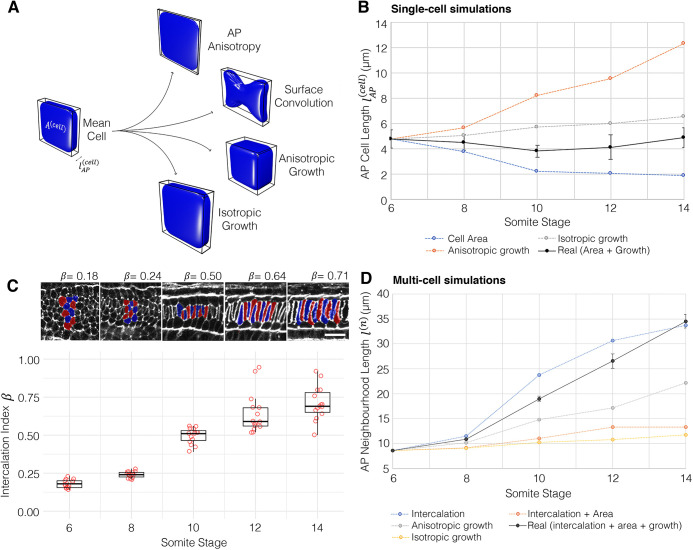
**Geometric modelling predicts contributions of shape and size to major transitions of the central cell trajectory.** (A) Visual renderings of transformations identified in the central cell trajectory. (B) Results of geometric perturbations applied to a mean central layer cell at 6 ss, over a developmental time course, illustrating change in AP cell length. Coloured lines represent modelled scenarios; black line represents real measured change (effect of growth and cross-sectional area combined) ±1 s.e.m. (C) Calculations of intercalation index (β) across developmental time, with panels showing representative groups of 10 cells per stage. Boxes show the median, 25th and 75th percentiles. Whiskers show maximum and minimum values within 1.5 times the interquartile range. *n=*15/stage. Scale bar: 20 µm. (D) Predicted changes in length of a neighbourhood of 10 6 ss central layer cells. Black line shows real measured length changes ±s.e.m., coloured lines show modelled scenarios. Mean cells calculated from *n*=344.

By deconstructing notochord development in morphospace, we have shown that AP anisotropy, the DV:ML ratio, growth and surface convolution are key geometric transformations underpinning central cell differentiation (Figs 2 and 3A). To investigate the relative contributions of each of these transformations to shape changes in single cells, and groups of intercalating cells, we set out to model the effects of each transformation when acting independently. To this end, we devised a simplified mathematical framework to model the effect of each transformation on a mean cell at 6 ss, when acting alone (see supplementary Materials and Methods). This mean cell is defined by its AP length 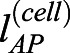, volume *V*^(*cell*)^ and transverse cross-sectional area *A*^(*cell*)^. Where *s* denotes stage, AP length can be approximated as(1)
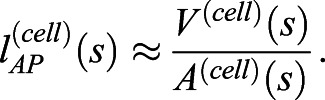
We can now measure change in cell length when volume or cross-sectional area change independently, with the other fixed at their 6 ss value.

As shown, central notochord cells in the 40-60% region undergo an early shortening on the AP axis linked to intercalation (ST1, 6-10 somites), followed by a late phase of elongation in which AP length is restored (ST2, 10-14 somites) (Fig. 3B black line). We first set out to investigate how cell length would change if governed only by AP anisotropy, in the absence of growth. Here, we allowed cell length to change in accordance with measured change in cross-sectional area, while maintaining the cell at constant volume (Fig. 3B, blue line). Under these conditions, cross-sectional area increases at the expense of length, and cells undergo a 54% shortening between 6 ss and 10 ss. Thereafter, instead of restoring length, cells continued to shorten by an additional 15%. This suggests that transverse cell spreading has a sustained negative effect on cell AP length. Without prior knowledge of growth dynamics, we envisaged two scenarios for its effect on shape. First, we considered a scenario in which growth acts isotropically, to expand cell geometry proportionally in all directions. In this case, cells elongated by 37%, exceeding the 2% net length change measured during normal development (Fig. 3B, grey line). This change was more pronounced in a second scenario of anisotropic growth, in which we prevented radial cell expansion and forced growth to act unidirectionally on AP length. In this case, we measured a 159% elongation (Fig. 3B, orange line), again exceeding the real measured change. Collectively, these calculations suggest that transverse cell spreading behaviour and growth make antagonistic contributions to cell AP length, and the real profile of length change is a dynamic balance between the two transformations: up to 10 ss, the rate of transverse cell spreading outweighs that of growth, leading to a net cell shortening during intercalation. When cross-sectional area stabilises at 10 ss (Fig. 2E), growth then dominates to translate a programme of transverse cell spreading into one of elongation.

We next sought to test how this relationship between shape and size in single cells affects the rate of elongation in a group of neighbouring cells undergoing intercalation. We tested this in groups of 10 cells from the 40-60% region. To investigate the effect of intercalation, we defined an intercalation factor, *β*, that allows calculation of extended AP group length *l*^(*n*)^ from cell number *n* and mean AP cell length 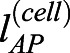 for a given stage (Fig. 3C, supplementary Materials and Methods):(2)
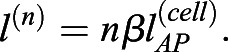
We used values for *l*^(*n*)^ measured for groups of 10 adjacent cells in the embryo at each stage. When *β*=1, group length equates to the summed lengths of all cells, whereas when *β*<1 cells are displaced from the midline, so their individual lengths are not additive in the same plane (Fig. 3C). We first tested how intercalation alone contributes to neighbourhood length by applying β values calculated at each stage to groups of mean 6 ss cells (Fig. 3C). Here, we found that intercalation can drive a 249% AP elongation, occurring at its greatest rate between 8 ss and 12 ss (Fig. 3D, blue line). This is the effect of simply aligning cells into a single-file array, with no accompanied shape change. To factor in cell shape changes, we applied measured changes in both cross-sectional area and *β*, while keeping volume constant (Fig. 3D, orange line). In this growth-free scenario, intercalation did increase neighbourhood length, despite the shortening of individual cells, but only by 55%, measured at 12 ss. This falls significantly short of real measured values, suggesting that the ability of intercalation to drive AP tissue elongation is counteracted by cell spreading behaviours that reduce cell length. We therefore hypothesised that growth should account for most of AP elongation. Alone, isometric growth achieved only a 76% elongation (Fig. 3D, yellow line), and anisotropic growth achieved a 159% increase (Fig. 3D, grey line). This means that growth is also insufficient to drive full tissue elongation, unless coupled to intercalation. Together, geometric modelling suggests that growth enables convergent extension in two manners. First, by counteracting loss of cell AP length during intercalation due to cell spreading (6-10 ss). Second, by further increasing cell length after intercalation, once the stack-of-coins is formed (10-14 ss).

### Spatial mapping reveals bi-directional gradients of shape maturation

**Fig. 4.**
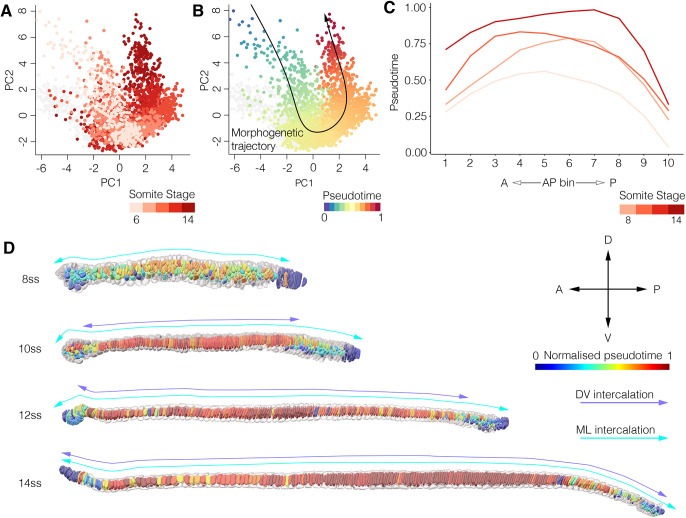
**Temporal variation is spatially organised in bi-directional gradients.** (A) All central layer notochord cells projected into morphospace, colour-coded for somite stage. (B) Central notochord cells ordered across an inferred pseudotemporal axis, guided by prior information on temporal transitions. (C) Mean pseudotemporal distance for cells in 10 evenly sized bins of the AP axis, in embryos between 8 ss and 14 ss. (D) Colour-coding of the central notochord layer by pseudotemporal distance, aligned with the progression of ML and DV intercalation events inferred from transitions in cell topology. Progress of ML and DV intercalation marked by cyan and purple bi-directional arrows. Representative of *n*=3 notochords/stage, *n*=2666 central layer cells.

Having previously identified heterogeneity in the extent of shape maturation for cells of the same somite stage (Fig. 1E), we next sought to test whether temporal variation is spatially organised. To this end, we isolated all cells in the central notochord layer, regardless of position, and inferred a pseudotemporal axis passing through all points. In this case, cells from successive somite stages were strongly overlapping in morphospace, although dispersal across the central cell trajectory increased progressively over time (Fig. 4A). The inferred trajectory initiated in the central branch of morphospace, passed through the bifurcation with Müller cells*,* and extended through the central cell branch (Fig. 4B). We next quantified spatial variation in pseudotemporal progression by calculating the mean pseudotemporal distance for 10 non-overlapping bins of normalised AP notochord length (Fig. 4C). Using this approach, we found for each stage that the most advanced cells in pseudotime concentrated in the middle of the notochord, and pseudotemporal distance progressively decreased towards the most anterior and posterior tips (Fig. 4C). When cells are colour-coded by AP position in morphospace, those at the leading edge of the central cell trajectory at each stage are derived from the pharyngeal region, whereas more immature morphologies correspond to increasingly posterior regions (Fig. S6A-D). Cells of the anterior tip also lie proximal to leading cells (Fig. S6A-D). At 10 ss, cells of the 0-15% region occupy the same position as 40-60% cells at 8 ss (Fig. S6A,B), and at 14 ss they overlap with 40-60% cells at 12 ss (Fig. S6C,D). In summary, this analysis reveals temporal variation in cell shape transitions to be spatially organised in bidirectional gradients across the AP axis.

To illustrate the spatial structure of temporal variation *in situ*, we colour-coded whole notochords by pseudotime (Fig. 4D). This confirmed the enrichment of leading cells in pseudotime in the middle part of the notochord, and a delay towards the anterior and posterior tips. It additionally revealed a correlation between the extent of shape maturation and changes in multi-cellular topology. Cells in the middle of the notochord were the first to undergo dorsoventral intercalation, as inferred from the local transition from a multi-layered organisation to a trilaminar organisation (Fig. 4D, 10 ss). Unlike the 40-60% level, which undergo DV intercalation between 6 ss and 10 ss (Fig. 2I), cells in the anterior tip remained stratified on the dorsoventral axis until 10 ss and reduced to a single-layered organisation by 14 ss (Fig. 4D, 10-14 ss*,* Fig. S6E). This represents a four-somite delay compared with the trunk region. We additionally found the posterior notochord to exhibit delayed intercalation. At all stages analysed, progenitors in the most-posterior notochord remained stratified on the dorsoventral axis (Fig. 4D, Fig. S6E)*.* Up to 12 ss, we also found the persistence of tall columnar cells lacking bipolar left-right contacts at the extreme posterior tip of the notochord (Fig. 4D, Fig. S6E), characteristic of the monolayered archenteron roof prior to mediolateral cell intercalation (Fig. S1B, 3 ss). Over developmental time, cells in both of these topologies were depleted and restricted further towards the posterior tip of the embryo (Fig. 4D, Fig. S6E). In summary, the middle-to-tips dynamic of cell shape maturation in the notochord spatially and temporally mirrors bidirectional waves of ML and DV intercalation that establish a distinctive ‘stack-of-coins’ pattern in the central notochord layer.

### Spatial variants in trajectory structure demonstrate divergent and convergent paths to specific cell morphologies

**Fig. 5.**
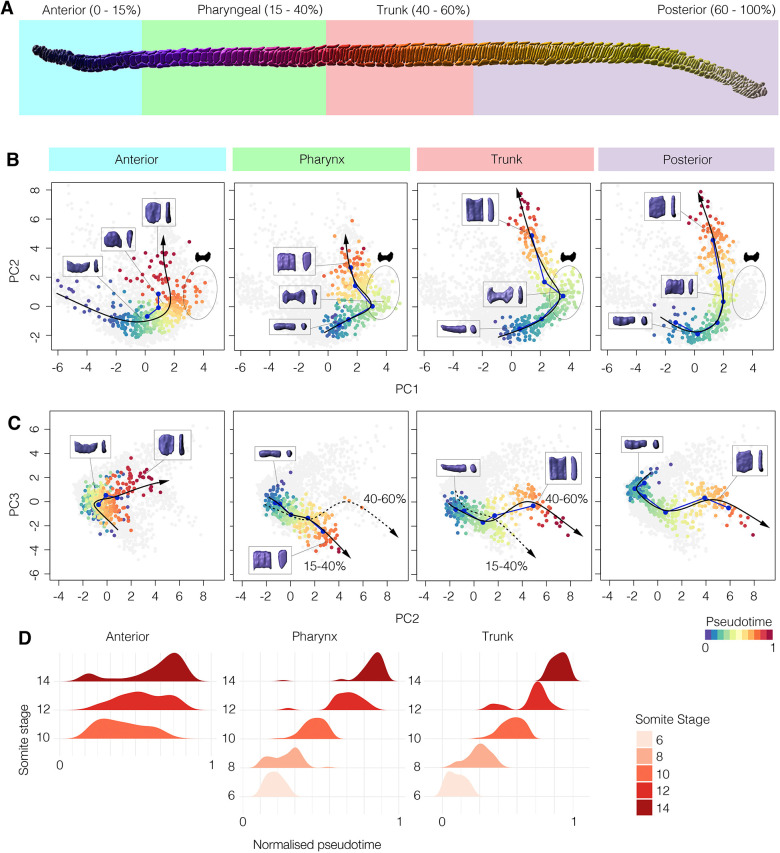
**Variation in cell shape trajectories across the AP axis.** (A) Segmented 14 ss notochord showing sampled regions on the AP axis for trajectory comparison. (B) Cells from each region defined in A projected on PC1 and PC2, with a segmented line connecting point cloud centres for each stage (blue) and an inferred pseudotemporal trajectory (black). For the posterior trajectory, six bins of AP length at 14 ss are used instead of somite stage. Pseudotemporal position is colour coded. (C) Projections of the same cells as B, against PC2 and PC3. For the 15-40% and 40-60% levels, both trajectories are highlighted for comparison. Representative cells are shown in insets; anterior (left) and lateral (right) views. (D) Density plots showing the frequency of cells across pseudotime for each somite stage in the anterior, pharyngeal and trunk regions. Anterior, *n*=396; pharynx, *n*=360; trunk, *n*=344; posterior, *n*=408.

Having identified spatial variation in developmental timing across the AP axis, we sought to test whether the structure of the central cell trajectory is shared by all cells, regardless of spatial position. We investigated this by constructing region-specific shape trajectories for four candidate regions along the AP axis (Fig. 5A). In each region, we performed trajectory inference as described previously. We then analysed variation between regions in both trajectory structure and its correlation with developmental time.

The trajectory of anterior cells involved similar geometric transitions to the trunk (compare Fig. 5B,C, anterior and trunk). This included a transient loss of AP length during intercalation (Fig. S7A), and net increases in cross-sectional area (Fig. S7D), volume (Fig. S7I) and DV:ML aspect ratio (Fig. S7L). Maturation additionally involved a decline in the coefficient of variation for cuboidness, although the mean values remained constant (Fig. S7G) and there was a progressive decline in sphericity (Fig. S7H). In accordance with its temporal delay compared with the pharynx and trunk, the anterior trajectory extended the least far into the central cell branch of morphospace (Fig. 5B,C). In addition, and unlike the more-posterior regions, there is a poor separation of cells over pseudotime between somite stages, reflecting loose temporal control of shape transitions in this region (Fig. 5D). When considered together, the anterior region shares common shape transitions to other regions, but is unique in its heterogeneity, with extensive variation in developmental timing between adjacent cells as they progress to their final morphologies.

The shape trajectories of the pharyngeal and trunk levels are similar to one another in that they display the two major transitions observed at the 40-60% level, including entry to the ‘bowtie domain’ at 10 ss (Fig. 5B,C, pharynx and trunk). In both cases, and unlike in the anterior region (Fig. 5B), cell shape changes were tightly controlled in developmental time (Fig. 5D). However, the pharyngeal trajectory was contracted on PC2 compared with the trunk trajectory, reflecting a lesser increase in the DV:ML ratio, and therefore a stabilisation of a square transverse profile rather than further elongating along the DV axis like cells in the trunk region (Fig. 5B*,* compare Fig. S5L and Fig. S8L). The pharyngeal trajectory also involves greater and more rapid increases in length and volume between 12 ss and 14 ss (compare Fig. S5A,I and Fig. S8A,I). Combined, these variations lead to a second bifurcation event in morphospace across PC2 and PC3 between 12 ss and 14 ss, yielding unique cell morphologies that are spatially resolved along the AP axis in the pharynx and trunk (Fig. 5C, pharynx and trunk).

To predict the trajectory of remaining posterior progenitors, which continue to differentiate after 14 ss, we used relative AP position to guide trajectory inference. This was justified by the correlation we identified previously between axial position and developmental maturity in the posterior region (Fig. 4C,D). In this region, cells were similar in initial and final morphologies to the trunk region, but appeared to circumvent the distinctive bowtie morphology, as shown by failure to populate the ‘bowtie domain’ occupied by pharyngeal and trunk cells at 10 ss (Fig. 5B, compare trunk and posterior). This implies a shortcut in their trajectories, such that maturation involves a continuous elevation of transverse area (increase in PC1) that is not enhanced by surface convolution. In summary, this analysis reveals discontinuity in shape transitions across the AP axis, in which some trajectories split over time to generate morphological diversity, while others take divergent paths to common forms.

### Posterior axial progenitors accelerate notochord elongation by increasing cell number

**Fig. 6.**
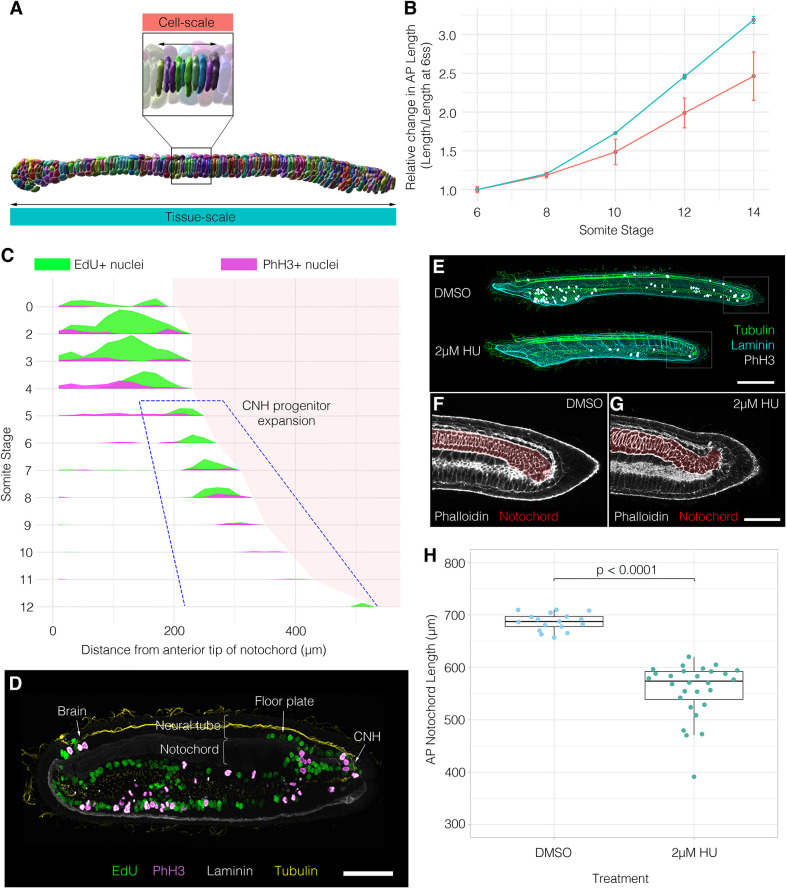
**Posterior addition is required for full extension of the notochord.** (A) 10 ss segmented notochord illustrating cell-scale (top) and tissue-scale (bottom) measurements of AP length. (B) Graph showing relative change in AP length at the cell scale (neighbourhoods of 10 adjacent cells, *n*=15 measurements/stage) and the tissue-scale (whole notochords, *n=*5 per stage) ±1 s.e.m. (C) Proliferation landscape for the notochord, showing mean frequency of EdU and PhH3 staining in embryos at successive somite stages, with length scaled to mean per stage (*n=*102 embryos). (D) Raw staining for EdU and PhH3 at 8 ss in a sagittal section, with tissue landmarks annotated. Anterior is leftwards; posterior is rightwards. (E) Effect of 2 µM HU between 6 ss and 14 ss on axial length. (F,G) Phalloidin staining in a sagittal section through tails of DMSO and HU-treated specimens. Notochord false-coloured in red. (H) Quantification of notochord length in DMSO and HU-treated specimens (*n*=17 DMSO, 27 HU). *P* value is result of unpaired two-tailed Student's *t*-test. Boxes show the median, 25th and 75th percentiles. Whiskers show maximum and minimum values within 1.5 times the interquartile range.

Our morphometric analysis decomposes notochord morphogenesis into a branching series of cell shape trajectories. Our final objective was to test whether the behaviours we identify at this scale are sufficient to account for shape change at the tissue scale. We therefore compared the dynamics of elongation at the neighbourhood level with that at the tissue level, using direct measurements of total notochord length (Fig. 6A). Here, we found the amount of whole-notochord elongation to exceed that of local cell neighbourhoods, owing to a specific acceleration in rate after 8 ss (Fig. 6B). This discrepancy indicated that another factor, apart from local change in cell shape and size, must be affecting the rate of tissue elongation. We therefore hypothesised that addition of new cells through cell division might be required to explain full notochord elongation at the tissue scale.

To test this hypothesis, we first mapped out cell division dynamics in the notochord during its elongation using markers for nuclei in two cell cycle phases: EdU to label cells passing through S phase, and immunostaining for phosphorylated histone 3 (PhH3) to mark mitotic cells (Fig. S9). By plotting changes in the distribution of EdU^+^ and PhH3^+^ nuclei over developmental time, we identified a transition from broad cell division throughout the notochord, to a specific domain at the posterior tip at the onset of AP elongation (Fig. 6C,D). Therefore, we can infer that cells developing along shape trajectories defined for the anterior, pharyngeal and trunk regions of the notochord are post-mitotic during tissue elongation, whereas the posterior trajectory is continually fuelled by cell division at least until 8 ss. To functionally test the contribution of posterior proliferative progenitors to tissue elongation, we treated embryos with hydroxyurea (HU) at 6 ss, when proliferation becomes restricted to the posterior tip. HU-treated embryos elongated, but to only 81% of their expected length by 14 ss (Fig. 6E-H). Although the HU treatment is systemic, disorganisation of the posterior notochord in HU-treated embryos suggests that a lack of proliferation in this region has contributed to loss of AP tissue length (Fig. 6F,G). However, we cannot rule out the possibility that proliferation in the ventral endoderm, which occurs throughout axial elongation, may also influence notochord length (Fig. 6D, Fig. S9). In summary, although notochord length is primarily generated by cell rearrangement and growth, these data hint at a further role for posterior notochord progenitors in providing the additional cellular material required for full tissue elongation.

## DISCUSSION

Here, we decompose the development of an entire tissue – the amphioxus notochord – by embedding it in a single-cell morphospace. In this environment, morphogenesis is unravelled into a branching portrait of shape differentiation, in which cells transit along trajectories specific to cell type to form a stereotypical diversity of morphologies. When spatially mapped in the embryo, these trajectories organise into temporal gradients of shape differentiation, which align with stepwise changes in multicellular topology. Our approach exposes both stereotyped variation in cell morphology across the AP axis, and the convergence of cells on common morphologies through variable morphogenetic routes. Furthermore, because our approach allows extraction of dynamic information from static imaging data, it enables the inference of cell behaviour where *in vivo* imaging is not yet possible. This makes the approach widely applicable in a range of non-model organisms where genetic approaches and live imaging are challenging. Although the number of specimens that can be processed using manual segmentation is limited, these datasets can be used as ground truth to train automated segmentation approaches. This will allow our approach to scale up and to include different experimental conditions, further cell labelling approaches and data from different species.

Here, we have applied single-cell morphometrics to study notochord morphogenesis in the amphioxus. In doing so, we identify a core set of morphogenetic processes shared between cephalochordates and vertebrates, that may represent ancestral developmental principles.

An unexpected finding in our study was regional variation of cellular behaviour across the AP axis: in the anterior, we find a variable morphogenesis with asynchronous maturation between neighbours; in the pharynx and trunk, we find a unique dynamic in which a bowtie morphology disproportionately expands the cell-spreading area during intercalation; and in the posterior, we find a simplified shape trajectory fuelled by cell division. These variations may arise due to a common programme of differentiation occurring under unique mechanical conditions or be the effect of region-specific signalling and genetic regulation. This is supported by the explicit notochord regionalisation found in vertebrates. In the mouse, live imaging has revealed marked differences in morphogenesis between the anterior head process (AHP), trunk and tail notochord (Yamanaka et al., 2007). Unlike the trunk, AHP progenitors derive from the early- and mid-gastrula organiser without passing through the node (Kinder et al., 2001; Yamanaka et al., 2007). In contrast, the tail progenitors are unique in their active migration to move posteriorly to the node (Yamanaka et al., 2007). Another example of regionalisation is the vertebrate prechordal plate (PrCP), which derives from the most anterior axial mesoderm through anteriorly-directed cell migration (Tada and Heisenberg, 2012). Evidence for regionalised behaviour aligns with nested expression of Hox genes in the vertebrate notochord (Prince et al., 1998). Previous work has also identified regionalised gene expression in the amphioxus notochord, and the data presented here adds to this in hinting at readout of these variations as discrete cell behaviours (Albuixech-Crespo et al., 2017). In evolution, the striking variations in vertebrates may have emerged within semi-discrete morphogenetic fields that were already defined in the first chordates, manifest here in the amphioxus. Future work is now required to define the molecular and mechanical cues instructing each behaviour, and to define notochord sub-regions with greater spatial precision.

By deconstructing and reconstructing the trajectory for central notochord cells, we elucidate a balance of cell shape, size and topology that dictates the length of small cell neighbourhoods. First, we infer that ML intercalation does not contribute directly to notochord elongation. Rather, it drives a convergent thickening that increases the number of cell layers on the DV axis. This multi-layered organisation is then reduced to a trilaminar pattern through dorsoventral intercalation, linked to convergent extension and to tissue elongation. In this second process, individual notochord cells increase their transverse spreading area at the expense of their AP length. While transverse cell spreading may facilitate intercalation between neighbouring cells, our geometric modelling suggests that the coupled loss of cell length abrogates the contribution of intercalation to tissue elongation. In turn, we find that cell growth is required to counterbalance loss of cell length. As a result, we predict that cell growth is required in this system to ensure tissue elongation. During intercalation, cell growth buffers the loss of cell AP length due to cell spreading, thereby enabling cell intercalation to generate tissue length. After intercalation, cell growth further increases cell length, once cross-sectional area is stabilised, and its impact on tissue length is enhanced by formation of the ‘stack-of-coins’ pattern. This enables tissue length to further increase without any ongoing changes in cell topology. At this scale, we therefore predict that a tight spatiotemporal coordination of cell growth is required in the notochord to control tissue shape and size. In turn, the effect of growth on form is influenced by changes in cell shape and topology.

Our investigation also revealed an important role for cell division in posterior axial progenitors for defining notochord length at full extension. Indeed, neighbourhood elongation dynamics alone are insufficient to account for those at the tissue scale. We show that this discrepancy may be explained by cell division during notochord elongation, which generates cells for its posterior 20%. The precise contribution of notochord progenitors to AP tissue length needs to be experimentally assessed by localised perturbations of cell division. Importantly, the programme of cell shape morphogenesis that generates length operates only after cell division arrest, when waves of intercalation and growth propagate across the tissue. As such, cell division, intercalation and growth are temporally separated, but act cumulatively to generate tissue length. The amount of cell division we find in amphioxus, and its contribution to length, is relatively small compared with vertebrates (Bénazéraf et al., 2017; Steventon et al., 2016). However, its presence in amphioxus is important in offering an evolvable node for evolutionary change. This is supported by an increase in the role of cell division in notochord elongation throughout chordate evolution, with a minimal contribution in amphioxus and the greatest contributions in amniotes (Catala et al., 1996; Krol et al., 2011; Mugele et al., 2018). Ascidians are an exception, where no proliferation occurs during notochord elongation (Jiang and Smith, 2007). Given our results in amphioxus, the lack of proliferation in ascidians is likely to be a secondary loss. Altogether, our data suggest that the role of posterior proliferation is not inherently novel to vertebrates, rather it has arisen through changes in the magnitude of a cell division dynamic already at play in the first chordates.

The approaches presented here offer a new way of seeing in the study of tissue morphogenesis, one that enables holistic analysis of cell behaviours defining tissue geometry. In this case study, we use single-cell morphometrics to define notochord morphogenesis in the amphioxus, and in doing so shine light on principles of morphogenesis at the base of the chordate phylum. We find a conserved role for cell intercalation and growth in generating notochord length that complements previous studies in vertebrates, and also identify a number of evolvable nodes that predict the diversity of developmental dynamics found in vertebrate model systems. This includes evidence for spatial variation in developmental timing and regionalisation of cell behaviour across the AP axis, and a role for axial progenitor cells in accelerating notochord elongation through cell division. As a result, we propose that the diversification of notochord form and developmental dynamics in vertebrates has depended more on tweaks in the magnitude of morphogenetic processes already present in the first chordates, rather than their innovation *de novo*.

## MATERIALS AND METHODS

### Animal husbandry, spawning and fixation

Wild catch collections of amphioxus, *Branchiostoma lanceolatum*, were made in Banyuls-sur-Mer, France, and transported to a custom-made amphioxus facility in Cambridge, UK. Adult amphioxus were maintained and bred, and the progeny raised as described by Benito-Gutiérrez et al. (2013). All embryos were fixed in 3.7% PFA+MOPS buffer for 12 h, then stored in sodium phosphate-buffered saline (PBS)+0.1% sodium azide at 4°C.

### Embryo staining and imaging

Embryos were first permeabilised overnight in PBS+1% DMSO+1% Triton. They were then blocked in PBS+0.1% Triton+0.1% BSA+5% NGS and incubated overnight in primary antibodies as follows: rabbit anti-laminin (Sigma, L9393) at 1:50, rabbit anti-PhH3 (Abcam, ab5176) at 1:500 and mouse anti-acetylated tubulin (T 6793, Sigma) at 1:250.

The primary wash was performed in PBS+0.1% Triton+0.1% BSA, before a secondary block in PBS+0.1% Triton+0.1% BSA+5% NGS, and overnight incubation in goat anti-rabbit (Abcam, ab150083) and/or goat anti-mouse (Invitrogen, 84540) secondary antibodies at 1:250. Staining with DAPI at 1:500 and rhodamine phalloidin at 1:250 was performed with the secondary incubation. Embryos were washed thoroughly with PBS+0.1% Triton and mounted for confocal imaging on glass-bottomed dishes in 80% glycerol. All imaging was performed on an Olympus V3000 inverted confocal microscope at 30× optical magnification.

For EdU labelling, EdU was applied to live embryos in seawater at a final concentration of 20 µM for 2 h prior to fixation. Fluorescent detection of incorporated EdU was performed following the manufacturer's instructions using a Click-it EdU Alexa Fluor 647 Imaging Kit (Invitrogen) prior to primary antibody incubation.

### Drug treatment

Live amphioxus embryos were treated with 2 µM hydroxyurea (HU) (Sigma, H8627) or dimethylsulfoxide (DMSO; Sigma, 276855) continuously between 6 ss and 14 ss (18-34 hpf at 21°C). They were then fixed immediately for imaging.

### Cell segmentation and morphospatial embedding pipeline

#### Manual cell segmentation

*Z*-stacks of embryos immunostained for tubulin and laminin, and stained for actin with phalloidin, were imported to Imaris 9.2.1 for segmentation. The free-rotate tool was used to align the embryonic axes with the *xyz* coordinate system (AP∼*x*, DV∼*y*, ML∼*z*). See Fig. 1A for embryos stained as described.

Cells were segmented manually, using phalloidin staining to delineate cell outlines. 2D splines were drawn around each phalloidin-stained cell every two slices in parasagittal section, and assembled into 3D triangle meshes without smoothing (preserve detail option ticked). See Fig. 1B for segmented notochords, and Fig. 1D for single segmented cells.

Cell surfaces were validated by overlaying a false-coloured volume for each cell over the raw phalloidin signal in 2D sections. Any volume-phalloidin mismatches were manually corrected by re-segmenting the corresponding cells.

Cells were segmented in dense groups in the 0-15%, 15-40%, 40-60% and 60-100% levels. See Fig. 1B for segmented notochords, and Fig. 2I for further groups of cells segmented together.

#### Extraction of cell shape metrics

After cell segmentation, we exported the full suite of 3D shape/size/position metrics from Imaris in .csv format. This included:

(1) Raw AP/DV/ML position of centre of homogenous mass.
(2) 3D orientation of the major ellipsoid axis relative to the AP, DV, ML axes of the embryo. A value of 1 means the long axis is perfectly aligned with the specified axis (AP, DV or ML). A value of 0 means the long axis orients perpendicular to the specified axis.
(3) Surface area calculated as the ‘number of triangles’, of invariant size, fitting the surface mesh for a given cell.
(4) Dimensions of axis-aligned/object-oriented bounding boxes.
(5) Centre of homogenous mass for DAPI channel, masked in each cell surface.
(6) Prolate/oblate ellipticity.
(7) Volume.

For a comprehensive and unbiased sampling of cell shape parameters, further shape metrics were calculated to include those previously published (e.g. Tassy et al., 2006) and are stated in Table 1, where *l* represents length, *bb* refers to bounding box-derived metrics and *cell* refers to cell-derived metrics. A sample of shape metrics are illustrated in Fig. 1C.

**Table 1.**
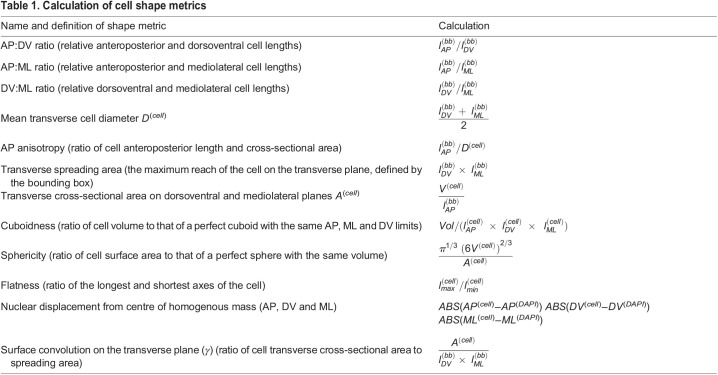
Calculation of cell shape metrics

#### Principal component analysis

Principal component analysis (PCA) was performed using the Factoextra package in R on all metrics describing qualitatively unique aspects of cell shape, size and orientation. See Fig. 1C for correlates of major components. We screened for redundant variables using the rcorr() function in the ‘HMisc’ package, and removed those describing common aspects of cell shape (e.g. ellipsoid axis lengths and true bounding box lengths) to prevent over-contribution to PCA eigenvectors. Spatial position and developmental stage were not included.

Metrics included in the PCA were:

(1) Angle of major ellipsoid axis relative to the AP, DV, ML axes of the embryo.
(2) Number of triangles.
(3) Volume.
(4) Relative AP, DV, ML dimensions (AP:DV, AP:ML, DV:ML ratios).
(5) AP anisotropy.
(6) Nuclear displacement from centre of homogenous mass on the AP, DV and ML axes.
(7) Cuboidness.
(8) Sphericity.
(9) Flatness.

The ‘scale’ function in Factoextra was used to standardise the data across all cells before calculation of principal components.

Individual shape features were considered to contribute significantly to a principal component if their contribution exceeded the mean contribution for all shape features.

PCA coordinates were later merged with the raw dataset to allow colour-coding and filtering for specific groups:

(1) Somite stage based on the number of somites counted along the AP axis using phalloidin, and laminin immunostaining (see Fig. 1E).
(2) Cell layer on the DV axis. Prospective Müller cells were filtered out as the most dorsal and ventral rows of notochord cells at each stage, regardless of morphology. All remaining cells were classified as ‘central’ layer.
(3) Position on the AP axis. We sampled four broad domains – the anterior (0-15% axial length), pharynx (15-40% axial length), trunk (40-60% axial length) and posterior (60-100% axial length) – where 0% is the anterior tip of the notochord and 100% is the posterior tip.

#### Morphospace embedding

Cells were embedded in morphospace by plotting them against pairs of PCs using ggplot2 in R. See Fig. 1D for total morphospace. Segmentation data for specific cells in morphospace was located using the live annotation function in Plotly to identify cell ID and embryo of origin. Images were then acquired in Imaris. See Fig. 1D (top panel) for morphospace with local cell sampling.

#### Morphogenetic trajectory inference

Trajectory inference was performed using the Slingshot package in R (Street et al., 2018), which uses clustering information on dimensionally reduced data to build a minimum spanning tree, and identify one or more principal curves. See Figs 2A and 5B,C for examples of inferred trajectories.

The data input to Slingshot was the coordinates of central layer notochord cells on the first three PCs. Somite stage was used as a clustering input to guide assembly of the minimum spanning tree. The start and end points of the trajectory were also defined as the first and final somite stages included in the analysis. This was performed individually for each region sampled on the AP axis.

For the posterior trajectory, clusters were defined as six bins of equal length across the AP axis of the 14 ss notochord. For trajectory inference regardless of AP position, k means clustering was used instead of somite stage.

For studying spatial variation, a single principal curve was identified for all regions, therefore representing a mean trajectory. See Fig. 4A,B for examples.

### Geometric modelling in cell groups and single cells

The effects of single geometric transformations on cell shape were simulated mathematically using a simplified parameterisation of cell shape at each somite stage. At each stage, we calculated mean AP cell length 
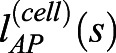
, volume *V*^(*cell*)^(*s*), transverse cross-sectional area *A*^(*cell*)^(*s*) and transverse spreading area, equating to the bounding-box cross-sectional area *A*^(*bb*)^(*s*). We then measured expected changes in AP cell length when specific transformations, corresponding to those measured between stages in real cells, occurred in isolation on mean 6 ss cells. Where *s* is the stage of interest and *s*_0_ is the starting stage of the experiment (6 ss), we measure the following:

AP anisotropy: change in 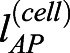 when *V*^(*cell*)^ remains at its 6 ss value and *A*^(*cell*)^ changes as normal, i.e.
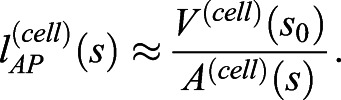
Isotropic growth: change in 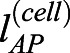 when *V*^(*cell*)^ changes as normal, and increases cell size proportionally in all directions, i.e.
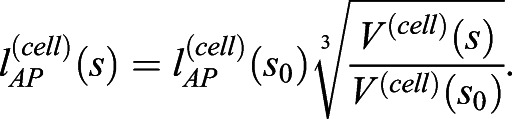
Anisotropic growth: change in 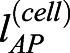 when *V*^(*cell*)^ changes as normal, but *A*^(*cell*)^ remains at its 6 ss value, so only affects 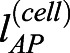, i.e.
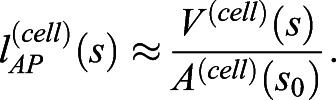

The lengths of cell neighbourhoods were calculated using the same calculations for cell-specific AP length, combined with a stage-specific intercalation correction factor, *β*(*s*). We experimentally measured *β* by dividing the real AP lengths of cell neighbourhoods in the notochord at each stage by the maximal elongation of a group of mean cells of the same number. The maximal elongation of a group of cells is defined as the neighbourhood length when cells are stacked perfectly on the AP axis in a ‘stack-of-coins’ topology, such that neighbourhood length equals 
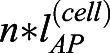
_._ The closer *β*(*s*) is to 1, the closer the cell group is to its maximal extension. Where *n* is cell number, we can now use *β*(*s*) and cell length *l*_*AP*_(*s*) to calculate neighbourhood length, *l*^(*n*)^(*s*), at a given stage,
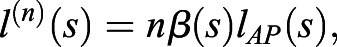
which allows us to uncouple contributions of intercalation and specific cell shape transitions to cell neighbourhood length. Full details and all equations used for geometric simulations are given in the supplementary Materials and Methods.

### Proliferation landscapes

To construct proliferation landscapes, the point selection tool in FIJI/ImageJ was used to identify a coordinate position along the AP axis for each nucleus positive for EdU or PHH3, and the most anterior and posterior nuclei in the embryo. The position for each labelled nucleus was then quantified across a normalised AP axis. Data were then pooled for embryos at each somite stage, and normalised length was scaled to the mean per stage. Mean frequency of EdU^+^ and PHH3^+^ nuclei was then plotted using the ggridges package in R to generate proliferation landscapes.

## Supplementary Material

Supplementary information
