# Supplementary material for: Single-cell morphometrics reveals ancestral principles of notochord development
Source: Development. 2021 Aug 19;148(16):dev199430. doi: 10.1242/dev.199430 (PMC8406538; doi:10.1242/dev.199430)
Supplement: Supplementary information [file develop-148-199430-s1.pdf]

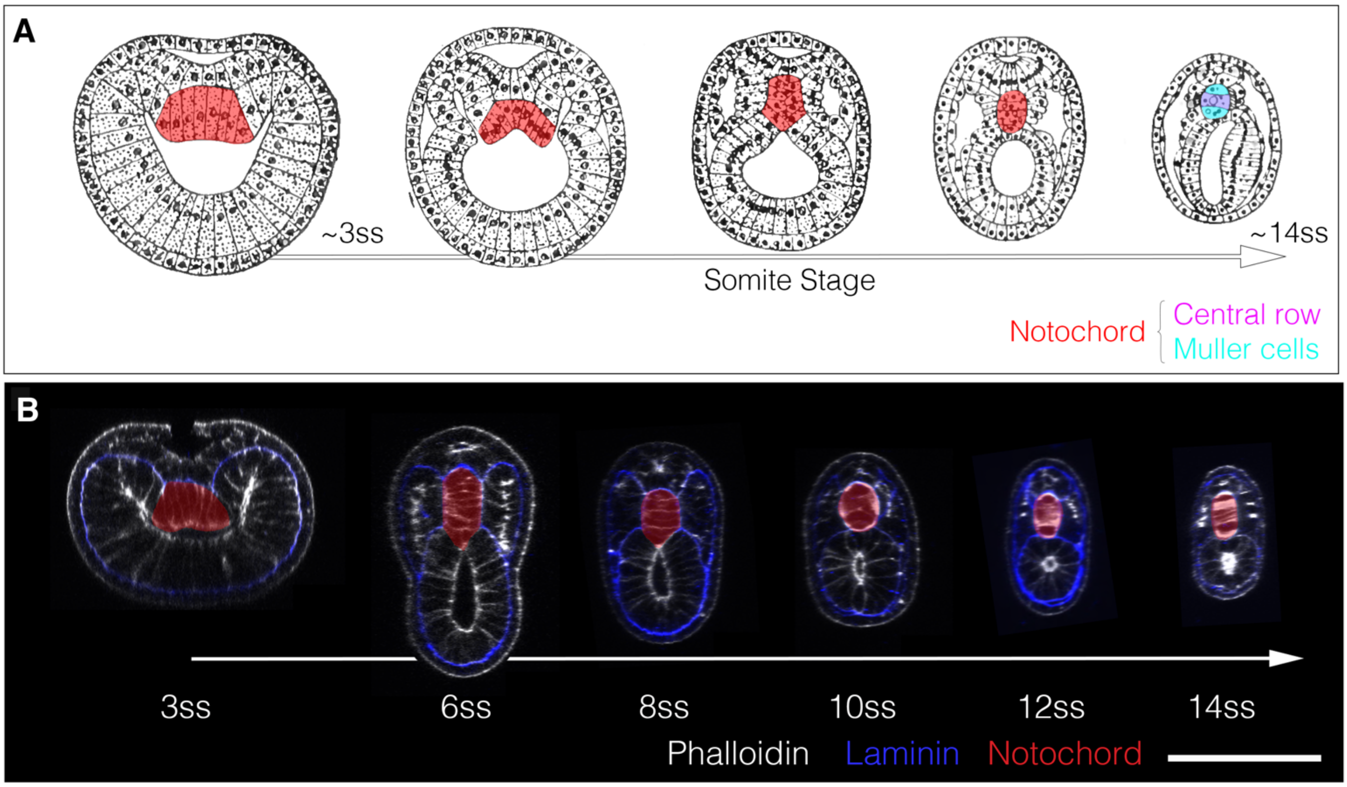

Fig. S1. Emergence of the amphioxus notochord from the archenteron roof. (A) Transverse sections of amphioxus embryos at successive somite stages, illustrated by Hatschek (1893). Coloured regions added by the authors show the changing position and cellular composition of the notochord as it evaginates from the archenteron roof and resolves into a trilaminar array on the DV axis. (B) Transverse sections of amphioxus embryos immunostained for laminin and stained with phalloidin and imaged with confocal microscopy over the same time course shown in (A). Notochord is false-coloured in red. Scale bar shows 50μm.

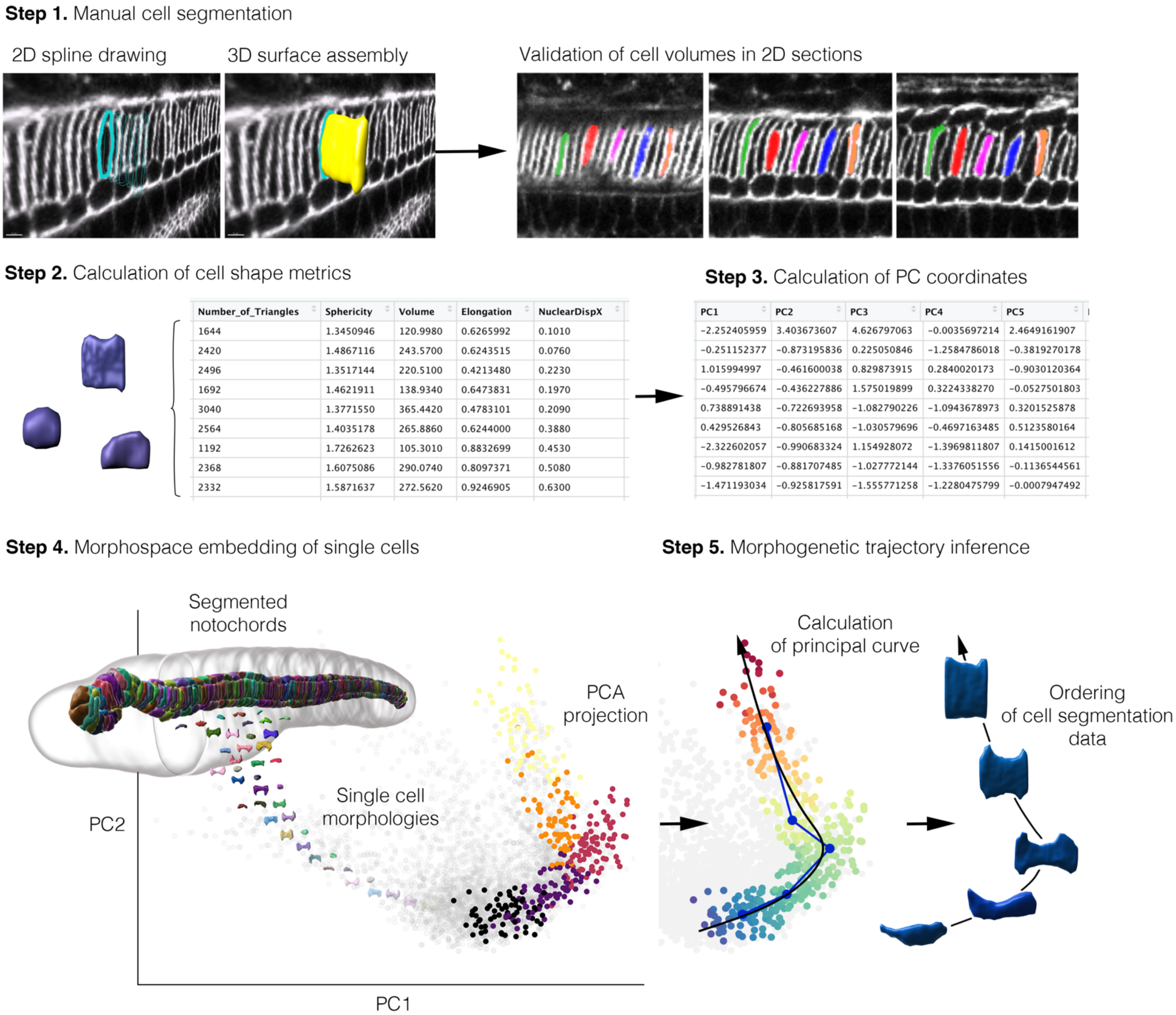

Number_of_Triangles	Sphericity	Volume	Elongation	NuclearDispX
1644	1.3450946	120.9980	0.6265992	0.1010
2420	1.4867116	243.5700	0.6243515	0.0760
2496	1.3517144	220.5100	0.4213480	0.2230
1692	1.4621911	138.9340	0.6473831	0.1970
3040	1.3771550	365.4420	0.4783101	0.2090
2564	1.4035178	265.8860	0.6244000	0.3880
1192	1.7262623	105.3010	0.8832699	0.4530
2368	1.6075086	290.0740	0.8097371	0.5080
2332	1.5871637	272.5620	0.9246905	0.6300

PC1	PC2	PC3	PC4	PC5
-2.252405959	3.403673607	4.626797063	-0.0035697214	2.4649161907
-0.251152377	-0.873195836	0.225050846	-1.2584786018	-0.3819270178
1.015994997	-0.461600038	0.829873915	0.2840020173	-0.9030120364
-0.495796674	-0.436227886	1.575019899	0.3224338270	-0.0527501803
0.738891438	-0.722693958	-1.082790226	-1.0943678973	0.3201525878
0.429526843	-0.805685168	-1.030579696	-0.4697163485	0.5123580164
-2.322602057	-0.990683324	1.154928072	-1.3969811807	0.1415001612
-0.982781807	-0.881707485	-1.027772144	-1.3376051556	-0.1136544561
-1.471193034	-0.925817591	-1.555771258	-1.2280475799	-0.0007947492

Fig. S2. Image segmentation and morphospace embedding pipeline. Panels illustrate key steps in the single-cell morphometrics pipeline used to infer cell shape trajectories in amphioxus notochord cells. See Materials and Methods section for further explanation of procedures involved in each step.

Development • Supplementary information

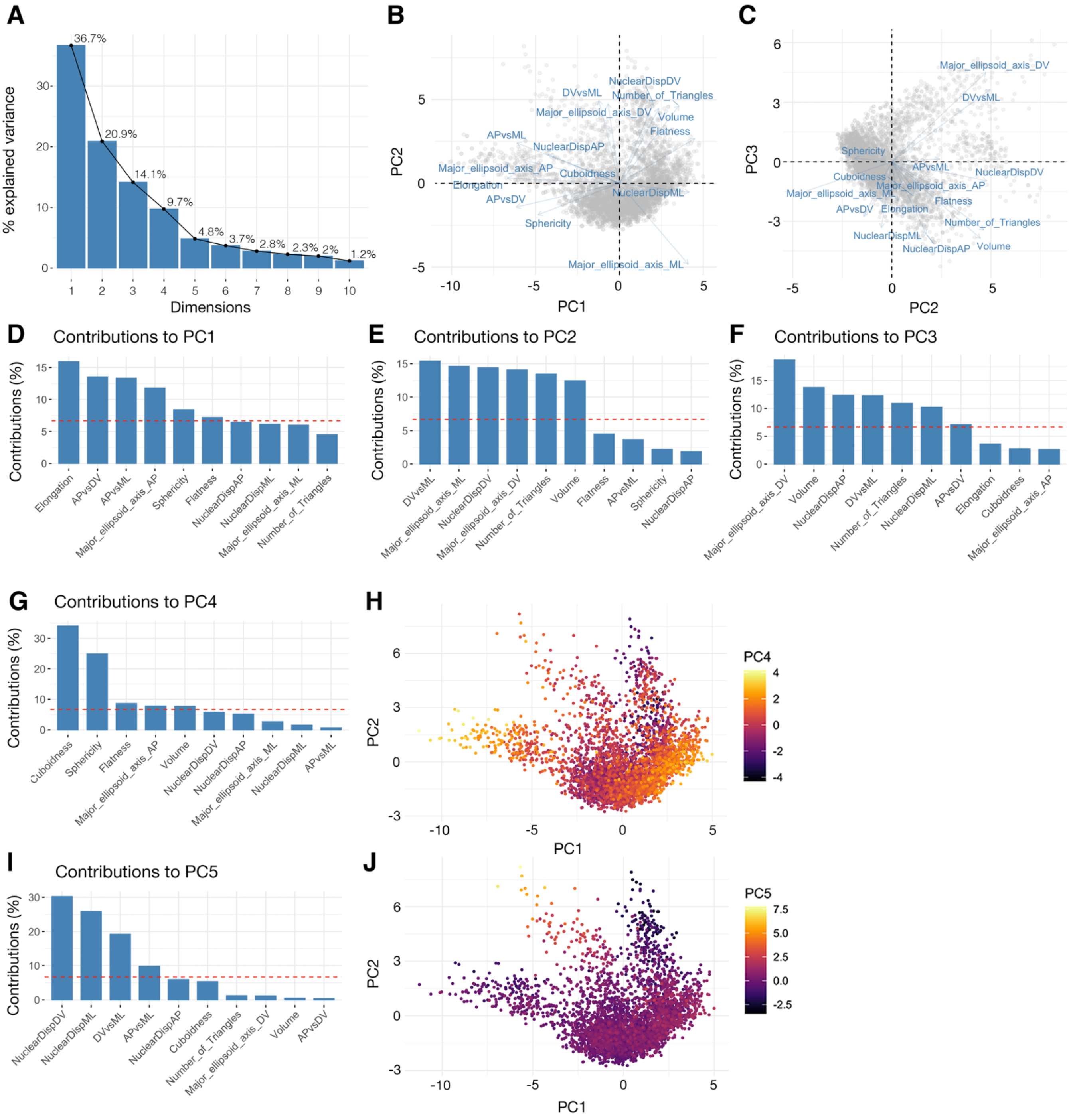

Fig. S3. Principal component analysis. (A) Scree plot showing contribution of eigenvectors to total dataset variation. First 5 eigenvectors account for 86.2% variation. (B, C) Compass plots showing direction of correlation between individual shape variables against PC1 and PC2 (B) and PC2 and PC3 (C). (D – I) Major geometric correlates of PC1 – PC5. Red dashed lines show the mean contributions of all shape variables. Variables exceeding this mean value are considered to make significant contributions to variation along the PC. (H) and (J) show all cell embedded in morphospace, plotted against PC1 and PC2, with colour code for PC4 (H) and PC5 (J). *n* = 3,796 cells.

Development • Supplementary information

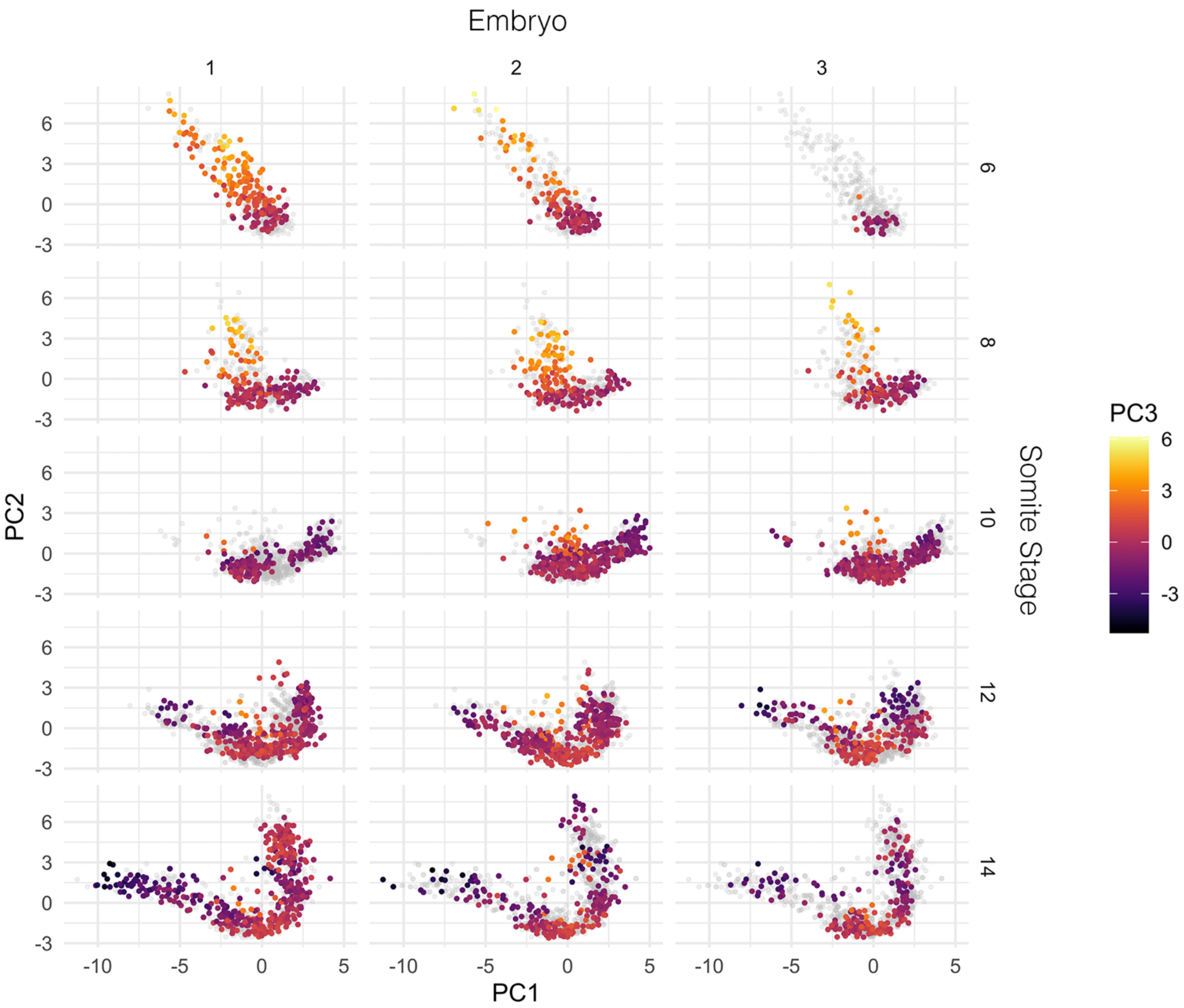

Fig. S4. Cell shape diversity is reproducible between different embryos. Graphs show all notochord cells embedded in morphospace against PC1 and PC2, filtered by embryo and somite stage. Grey points show all cells for the specified somite stage. Colour-code shows distribution of cells across PC3. *n* = 3,796 cells.

Development • Supplementary information

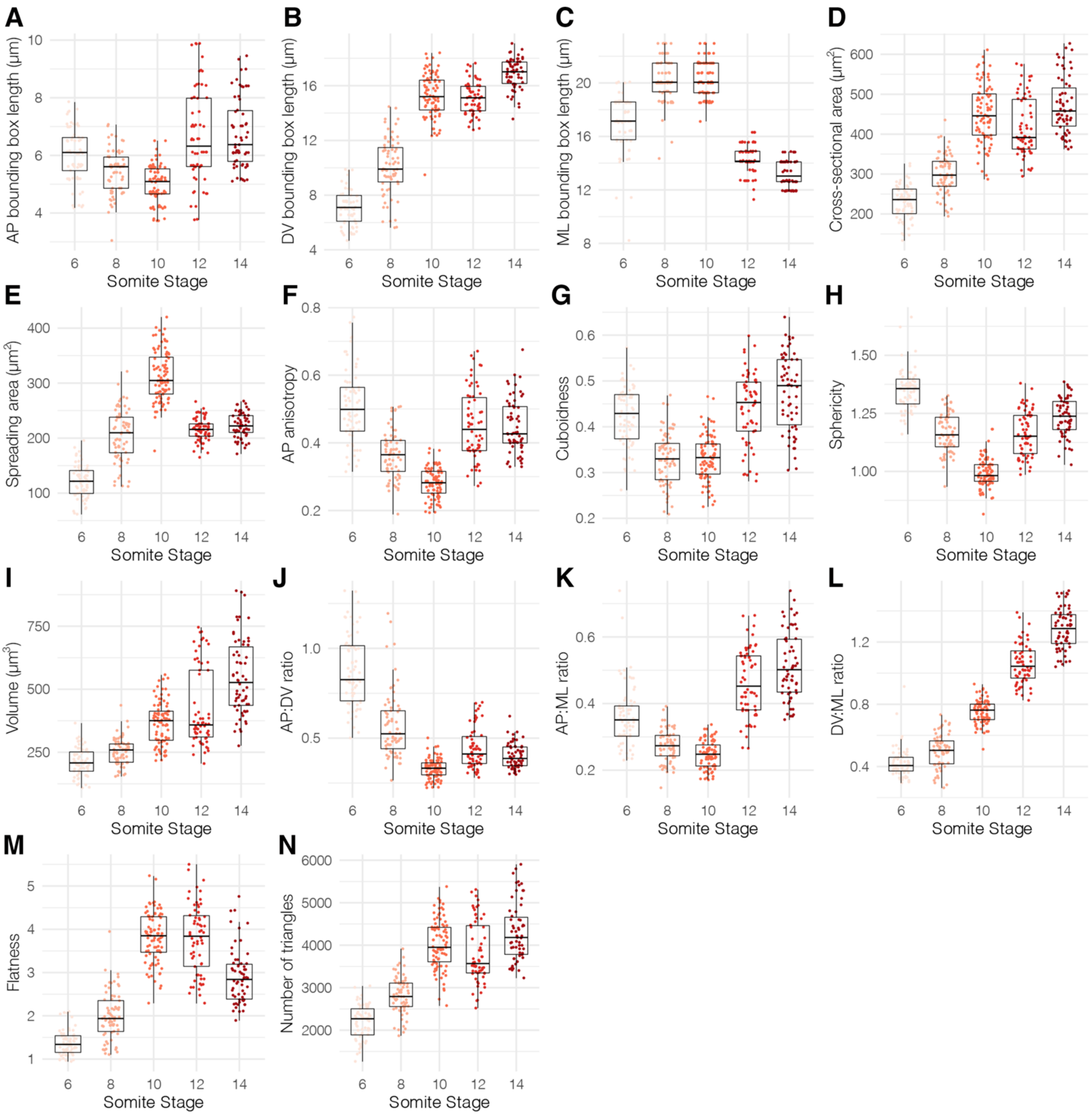

Fig. S5. Further geometric transformations in cells from the 40 – 60% AP level of the notochord. *n* = 344.

Development • Supplementary information

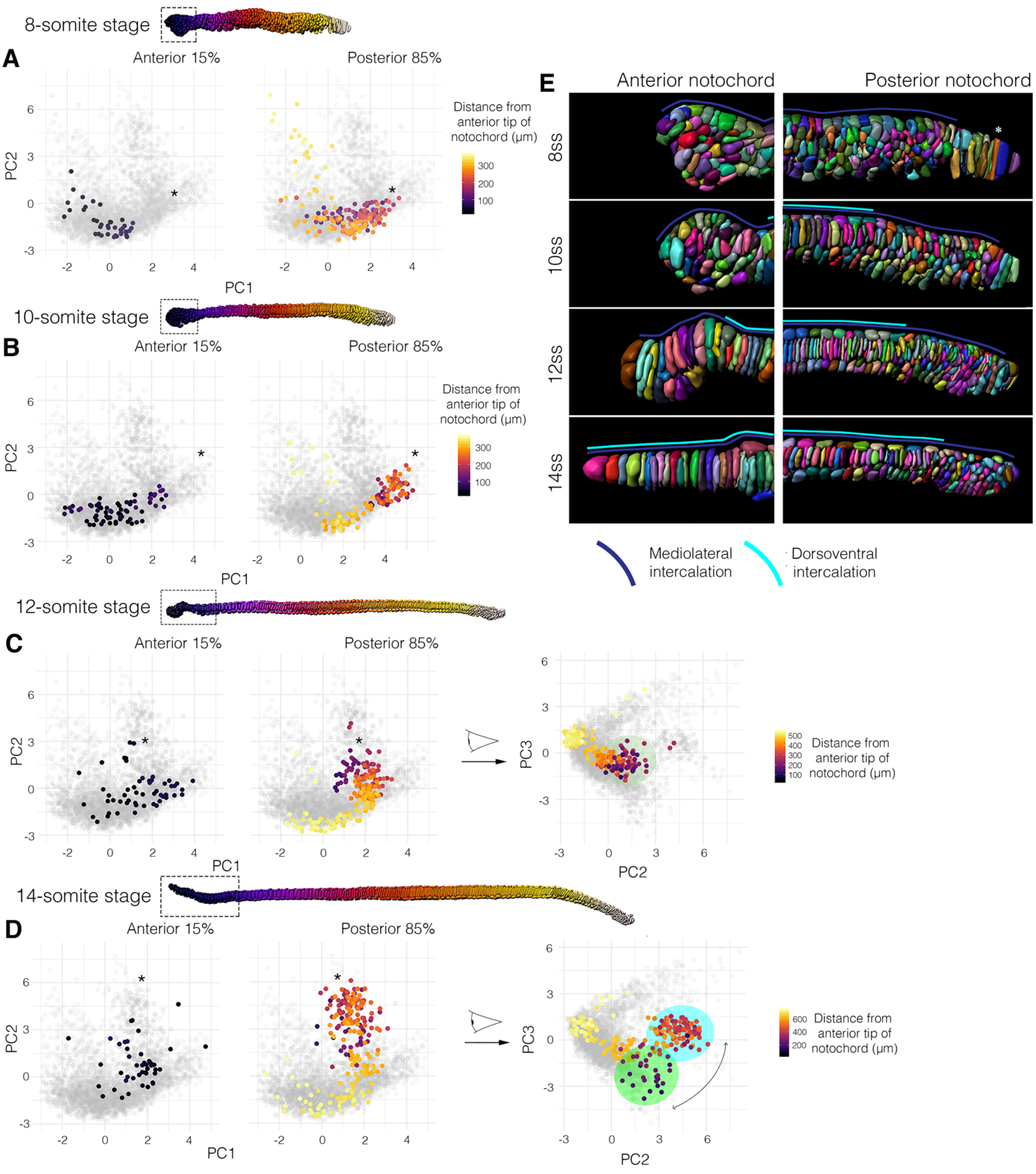

Fig. S6. Morphospatial embedding of whole notochords reveals spatial variation in developmental timing. (A – D) Central layer cells from whole notochords at 8ss (A), 10ss (B), 12ss (C) and 14ss (D) embedded in morphospace. Cells in the 0 – 15% AP region (dashed boxes over each notochord) and posterior 85% are plotted separately. Colour-code shows distance from anterior tip of notochord at each stage. In (C, D), cells of the posterior 85% notochord length are also shown against PC2 and PC3 (right panels), showing separation of two spatially-resolved populations between 12ss and 14ss. (E) View of segmented anterior (left) and posterior (right) tips of the notochord between 8ss and 14ss, with lines showing progression of ML and DV intercalation. n =2,866.

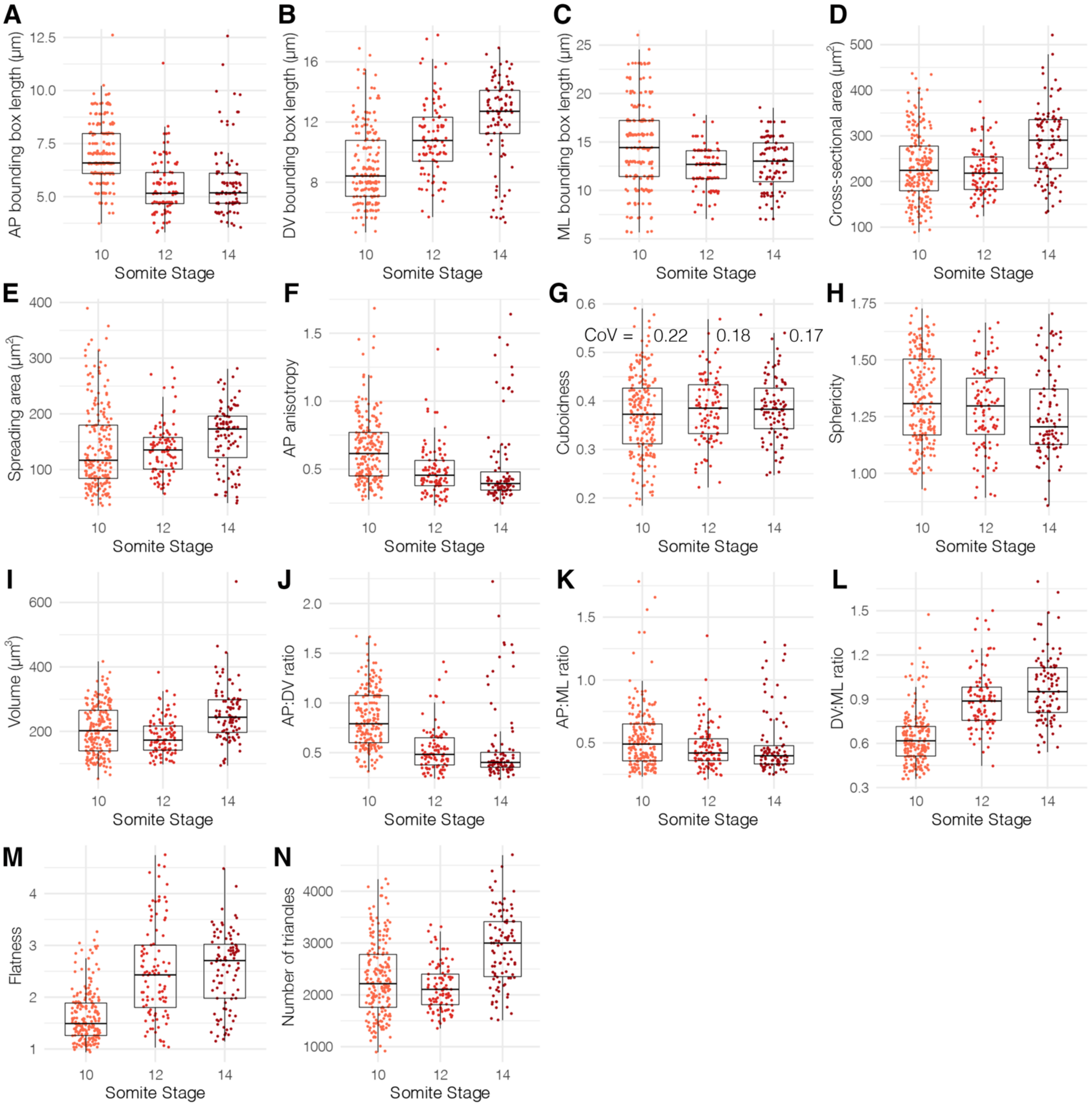

Fig. S7. Geometric transformations in cells from the 0 - 15% AP level of the notochord. CoV values denote coefficient of variation in panel (G). n = 396.

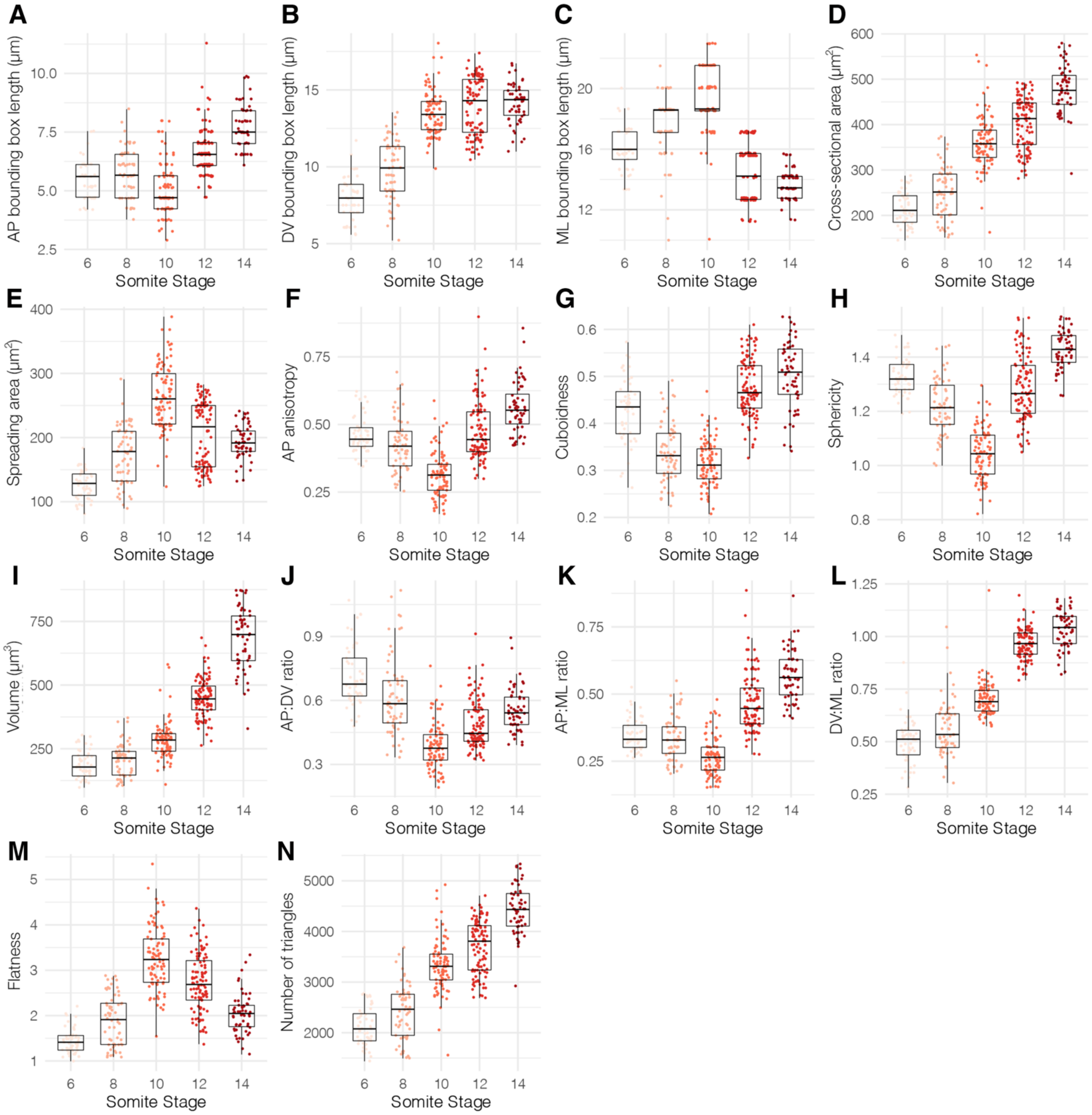

Fig. S8. Geometric transformations in cells from the 15 – 40% AP level of the notochord. *n* = 360.

Development • Supplementary information

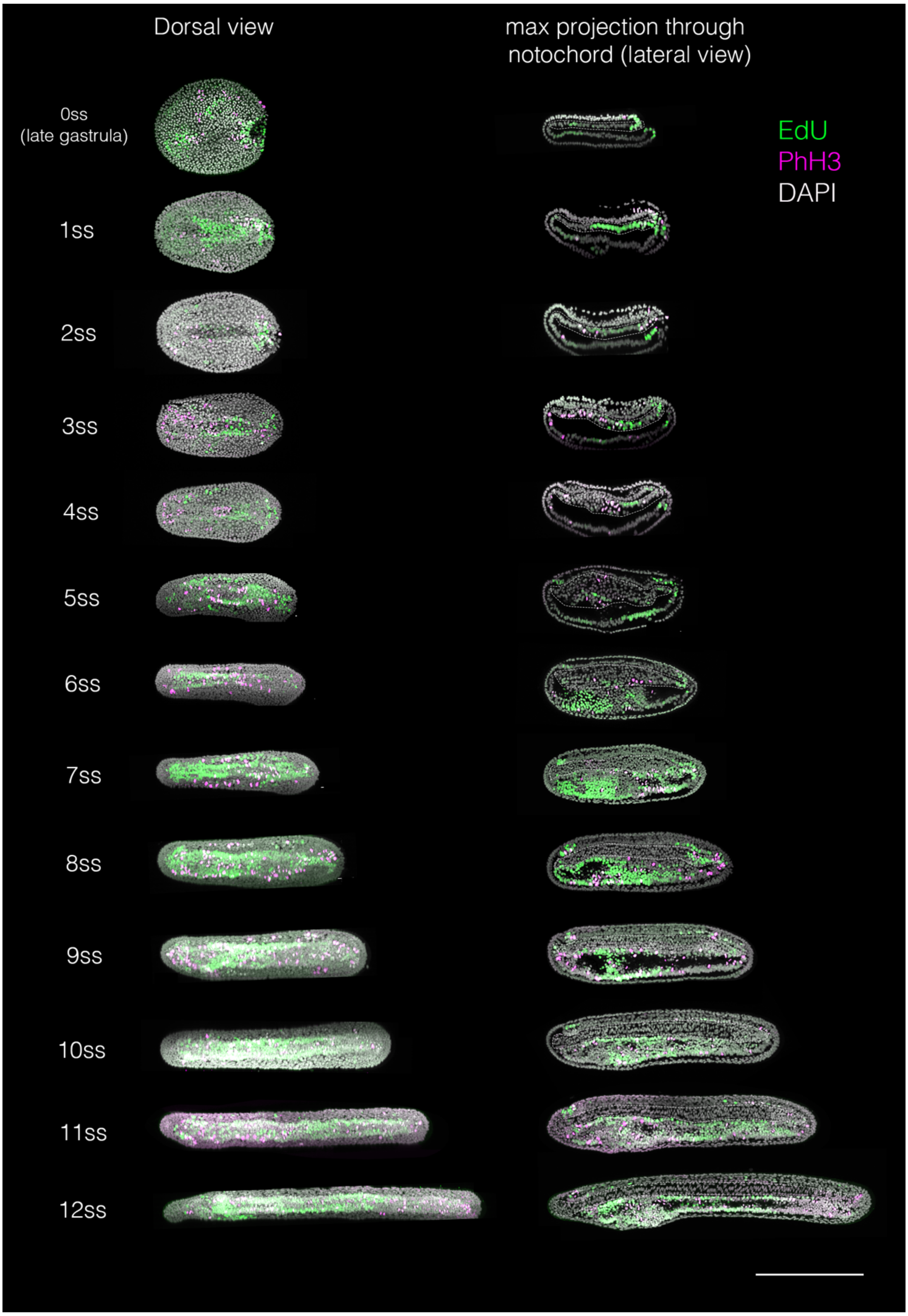

Fig. S9. Labelling of proliferative cells between gastrulation and 12ss. Embryos at successive somite stages were incubated in 20μM EdU for 2 hours prior to fixation (green), and immunostained for PhH3 (magenta) after fixation. For each stage, embryos are shown in maximal projection from a dorsal view (left) and in midline sagittal section (right). In sagittal sections, the position of the notochord is marked with a dashed white line. Scale bar shows 100μm. Representative of *n* = 102 embryos.

Development • Supplementary information

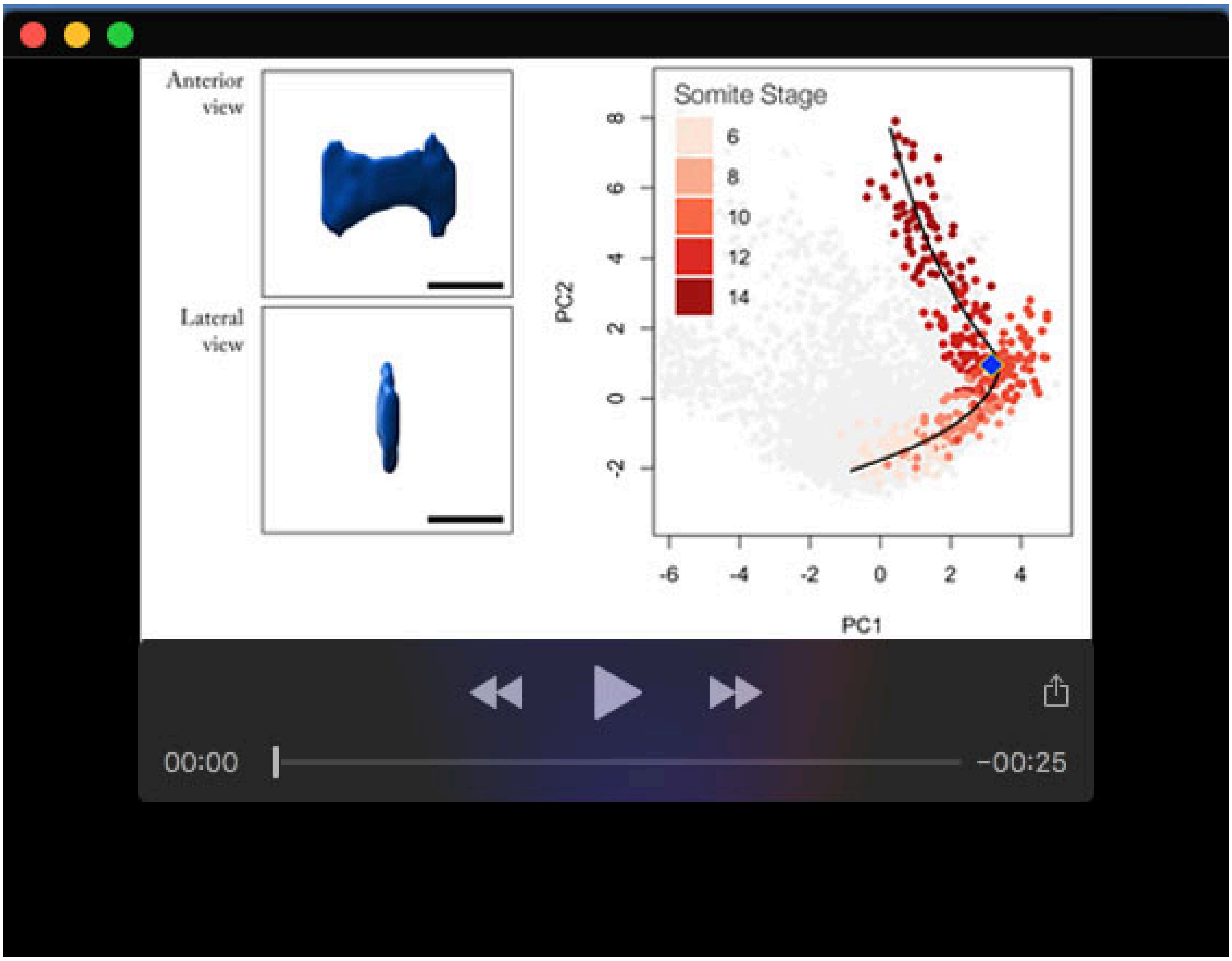

Movie 1. Cell shape transitions along the central cell trajectory. Video shows a sampling of cell segmentation data along the trajectory inferred for central layer cells at the 40-60% AP level of the notochord. (Left) Segmented cells in anterior and lateral views, as stated. (Right) All 40-60% central layer cells embedded in morphospace against PC1 and PC2, colour-coded by somite stage. Blue diamond shows position of cell illustrated in panels on left.

Supplementary Materials and Methods: Geometric Modelling

Geometric modelling in single cells

The aim of the geometric modelling is to identify how individual geometric transformations affect cell and tissue length in the notochord.

To achieve this, we first need to define a set of measures that allow for a simplified characterisation of the cell shapes and shape changes. To this aim, we first create an object-oriented bounding box around the cell, corresponding to the three-dimensional cell shape, and find that the box aligns with the anteroposterior (AP) axis, the dorsoventral (DV) axis and mediolateral (ML) axis of the embryo (Fig. S10). The bounding box has the volume $V^{(bb)}$ and is defined by three lengths, $l_{AP}^{(bb)}$, $l_{DV}^{(bb)}$ and $l_{ML}^{(bb)}$ (see Fig. 9B). We assume that the cells can be approximated as a two-dimensional shape that is oriented along the DV-ML plane and then projected along the AP axis for a length $l_{AP}^{(cell)}$ (see Fig. S10B and S10C). We find that bounding box length $l_{AP}^{(bb)}$ is a good approximation for cell length $l_{AP}^{(cell)}$, but convolution of the membrane on the transverse DV-ML plane generates a discrepancy between cell spreading area, defined by the bounding box area $A^{(bb)}$, and real cell area $A^{(bb)}$. In this case, we can assume

$$l_{AP}^{(cell)} \approx l_{AP}^{(bb)}.$$

We then define the spreading area

$$A^{(bb)} = l_{DV}^{(bb)} \cdot l_{ML}^{(bb)},$$

which corresponds to the transverse area (the area in the $DV - ML$ plane) of the bounding box, and estimate the transverse cross-sectional area of the cell,

$$A^{(cell)} \approx \frac{V^{(cell)}}{l_{AP}^{(cell)}},$$

where $V^{(cell)}$ is the cell volume. We also define the ratio

$$\gamma = \frac{A^{(cell)}}{A^{(bb)}},$$

which is a measure of how convoluted the transverse cell shape is in the DV-ML plane. For a small value of γ, the cell is characterised by one more long and thin elongations, generating a discrepancy between $A^{(cell)}$ and $A^{(bb)}$. For $\gamma = 1$ it fills the complete rectangle, such that $A^{(cell)}$ approaches $A^{(bb)}$ (see Fig. 9C).

We now start with mean cells at somite stages $s \in \{6,8,10,12,14\}$ which have the volume $V^{(cell)}(s)$, the anterior-posterior length $l_{AP}^{(cell)}(s)$ and the ratio $\gamma(s)$. These measurements are the

Development • Supplementary information

averages from all cells segmented from the 40 – 60% AP level of the notochord at each somite stage, including all three specimens segmented per stage.

From these measurements for mean cells we can calculate the spreading area as

$$A^{(bb)}(s) = \frac{A^{(cell)}(s)}{\gamma(s)} \approx \frac{V^{(cell)}(s)}{l_{AP}^{(cell)}(s)\,\gamma(s)}.$$

Alternatively, to calculate change in length $l_{AP}^{(cell)}$, we start with a cell of volume $V^{(cell)}(s)$, area $A^{(cell)}$ and ratio $\gamma(s)$. The length $l_{AP}^{(cell)}$ is now estimated by

$$l_{AP}^{(cell)}(s) \approx \frac{V^{(cell)}(s)}{A^{(cell)}(s)}.$$

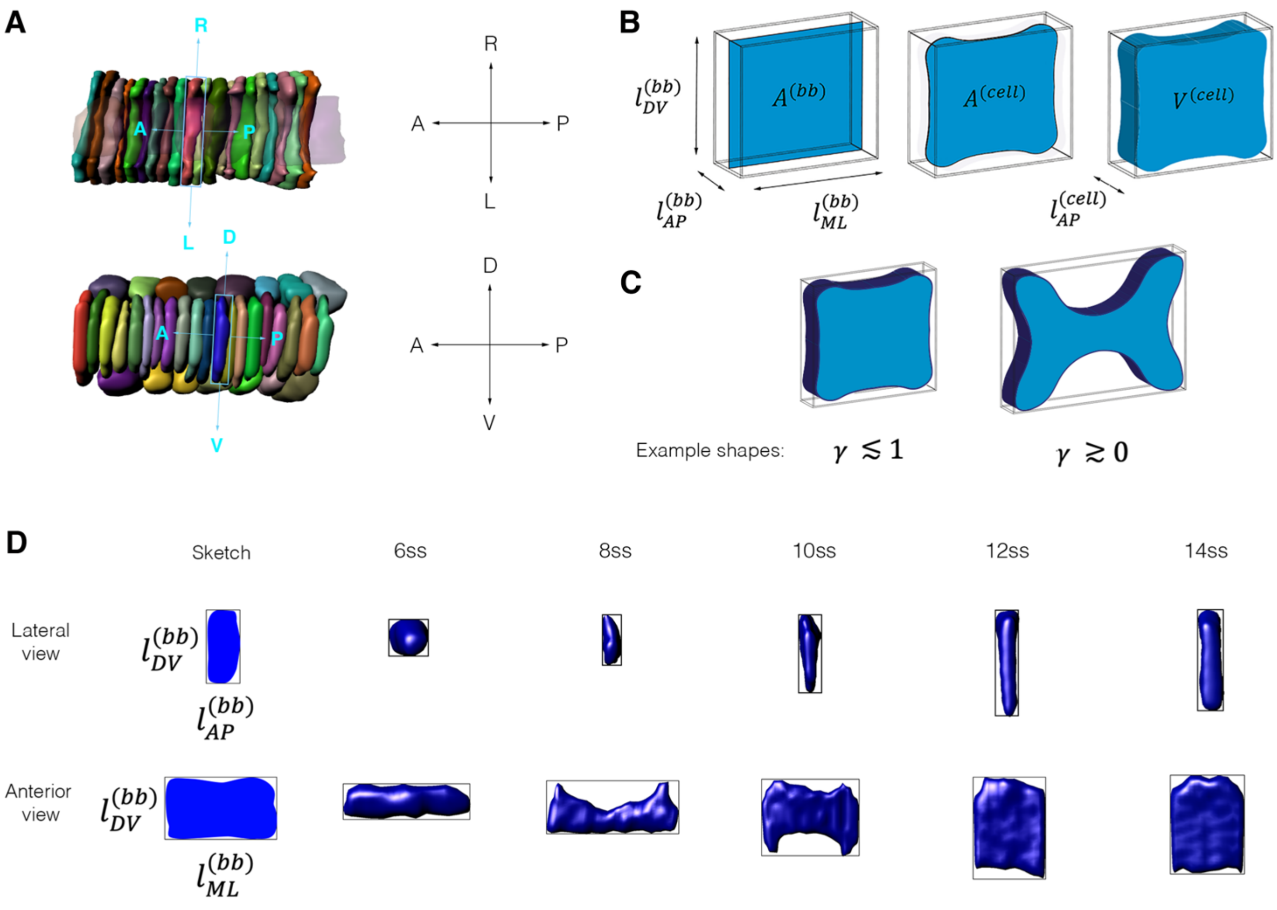

Fig. S10. Cell shape metrics used for geometric perturbations. (A) Object-oriented bounding boxes are calculated for each cell, and their axes are aligned to the AP, DV and ML axes of the embryo. (B) Schematised cells within object-oriented bounding boxes, showing the measurements of length, cross-sectional area and volume acquired. (C) Cells define the dimensions of their bounding boxes through variable degrees of surface convolution, which we quantify as γ. (D) Sample cells for each stage of development in lateral (top row) and anterior (bottom row) view within object-oriented bounding boxes.

Development • Supplementary information

The raw measurements used to define the mean cell at each stage are shown in Table. S1. The positions of cells in morphospace with mean values for all geometric parameters used for PCA are also shown in Fig. S11, within the total point cloud for the corresponding stage, with segmentation data for cells representing the extremes of shape variation for each stage.

Table S1. Geometries of mean cells used for geometric modelling

Stage (ss)	Volume (μm^3) ± s.d	AP length (μm) ± s.d	Transverse cross-sectional area (μm^2) ± s.d	γ ± s.d
6	211.49 ± 56.57	4.77 ± 0.73	44.34 ± 9.94	0.37 ± 0.059
8	251.48 ± 53.90	4.50 ± 0.60	55.88 ± 12.36	0.28 ± 0.050
10	365.18 ± 85.01	3.81 ± 0.49	95.85 ± 20.61	0.31 ± 0.047
12	422.94 ± 157.02	4.13 ± 0.98	102.41 ± 14.34	0.46 ± 0.053
14	547.84 ± 150.27	4.88 ± 0.80	112.36 ± 15.92	0.50 ± 0.062

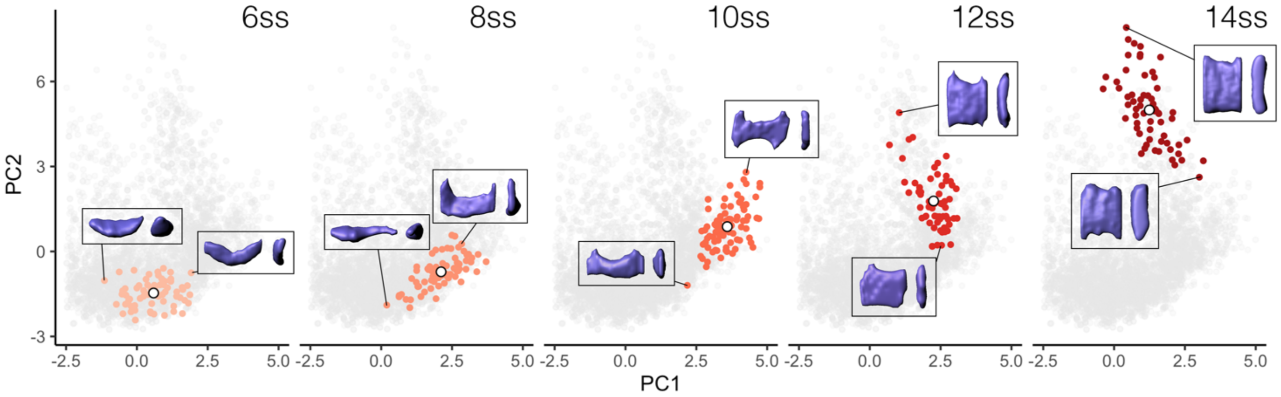

Fig. S11. Positions of 'mean cells' in morphospace for each somite stage. Central layer cells from the 40-60% AP level on the notochord filtered by somite stage, 6ss – 14ss. White points mark the locations of cells with mean values for all geometric parameters used for PCA, including volume, AP length, transverse cross-sectional area and γ. Inlays show segmented cells corresponding to specific points at the extremes of each point cloud, in anterior (left) and lateral (right) views. In each iteration of geometric modelling, we measure the effects of each of these transitions on AP length when acting in isolation. n = 344 cells.

We can now measure the effects of change in cell volume, transverse cross-sectional area (affecting the level of AP anisotropy) and surface convolution on cell spreading area and AP length using the equations shown in Table. S2. Here, s_0 defines the initial state of the cell, at 6ss. We find that the cell length along the AP axis only changes when the cell is elongated in this direction, grows anisotropically in this direction or isotropically in all directions. A convolution that does not affect the transverse cross-sectional area $A^{(cell)}(s)$ will also not affect the elongation.

Table S2. Equations applied for geometric modelling in single cells

**Transformation**	**Spreading area** $A^{(bb)}(s)$	$\boldsymbol{AP}$ **cell length** $l_{AP}^{(cell)}(s)$	**Sketch**
Change only in transverse cross sectional area $A^{(cell)}(s)$	$\frac{A^{(cell)}(s)}{\gamma(s_0)}$	$\frac{V^{(cell)}(s_0)}{A^{(cell)}(s)}$	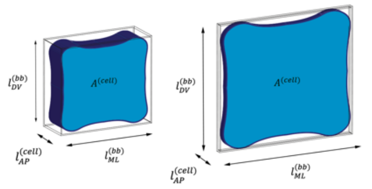
Isotropic growth $V^{(cell)}(s)$	$\frac{V^{(cell)}(s_0)}{l_{AP}^{(cell)}(s_0)\,\gamma(s_0)}\left(\frac{V^{(cell)}(s)}{V^{(cell)}(s_0)}\right)^{\frac{2}{3}}$	$l_{AP}^{(cell)}(s_0)\sqrt[3]{\frac{V^{(cell)}(s)}{V^{(cell)}(s_0)}}$	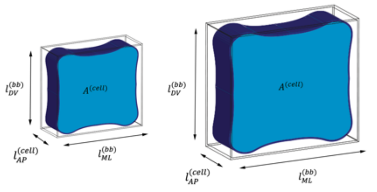
Anisotropic growth only along AP axis $l_{AP}^{(cell)}(s)$	$A^{(bb)}(s_0)$	$\frac{V^{(cell)}(s)}{A^{(cell)}(s_0)}$	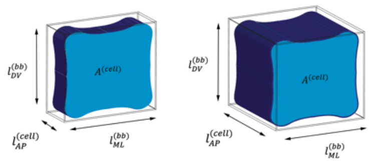
Convolution in the transverse plane	$\frac{V^{(cell)}(s_0)}{l_{AP}^{(cell)}(s_0)\,\gamma(s)}$	$l_{AP}^{(cell)}(s_0)$	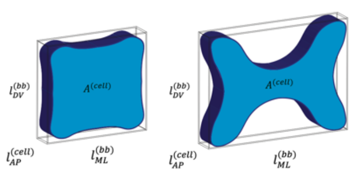

Development • Supplementary information

Geometric modelling in cell groups

To compute how these geometric transformations in single cells affect the length of a group of cells undergoing intercalation, we define a measure of cell intercalation at each stage. To this aim, we assume that the length contribution of a group of n cells (where we assume that n is large) in the AP direction is given by $l^{(n)}$ and the equation

$$l^{(n)}(s) = n\,\beta(s)\,l_{AP}(s),$$

with the intercalation correction $\beta(s)$. We calculate $\beta(s)$ at each stage as a ratio of cell neighbourhood length, $l^{(n)}(s)$, to the maximal possible extension of a group of cells of the same number. The maximal elongation of a group of cells is defined when cells are stacked perfectly on the AP axis in a stack-of-coins topology, and neighbourhood length equals $n\,l_{AP}^{(cell)}(s)$. $l^{(n)}(s)$ is manually measured in the notochord at each stage, as the AP length of groups of n cells measured in the 40-60% region. For our calculations of $\beta(s)$, n = 10. Cell groups were defined by selecting a cell at random, and identifying its nearest 9 cells in AP position. Where *n* is large, groups identified with this method include cells from all DV layers of the notochord, regardless of intercalation state. AP length of the group was then measured between the most anterior and posterior phalloidin signal using a straight line tool in FIJI. For $\beta = 1$, cells are perfectly stacked on the AP axis, whereas when $\beta \gtrsim 0$, many layers of cells are present on the DV plane (see Fig. S12).

We now start with a group of n cells, each of volume $V^{(cell)}(s)$, area $A^{(cell)}(s)$ and ratio $\gamma(s)$, and an intercalation level of $\beta(s)$, and calculate neighbourhood length as

$$l^{(n)}\,(s) \approx \; n\,\beta(s)\,\frac{V^{(cell)}(s)}{A^{(cell)}(s)}.$$

With this, we can study the effect of each geometric transformation in single cells, with and without intercalation, on neighbourhood length $l^{(n)}\,(s)$, using the equations shown in Table. S3.

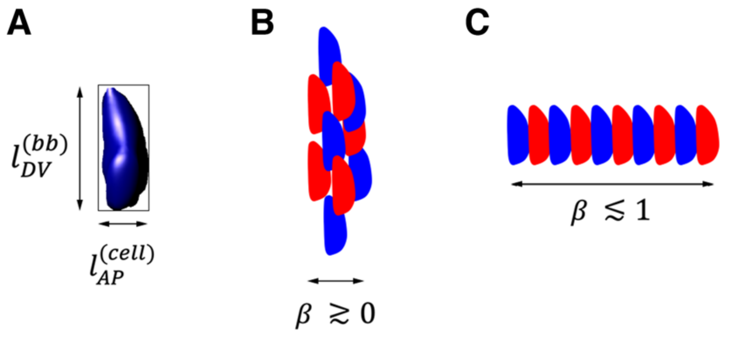

Fig. S12. Calculation of intercalation correction β in a neighbourhood of 10 cells. (A) Sample cell from 8ss in lateral view showing the direction of AP length $l_{AP}^{(cell)}$, approximated by $l_{AP}^{(bb)}$. (B, C) Schematic of a neighbourhood of 10 cells in lateral view, showing two extremes of β, approaching 0 in (B) and approaching 1 in (C).

Development • Supplementary information

Table S3. Equations applied for geometric modelling in cell neighbourhoods

<table>
<tr><th>Transformation</th><th>AP neighbourhood length
$l^{(n)}(s)$</th><th>Sketch</th></tr>
<tr><td>Intercalation only
$\beta(s)$</td><td>$n\,\beta(s)\frac{V^{(cell)}(s_0)}{A^{(cell)}(s_0)}$</td><td>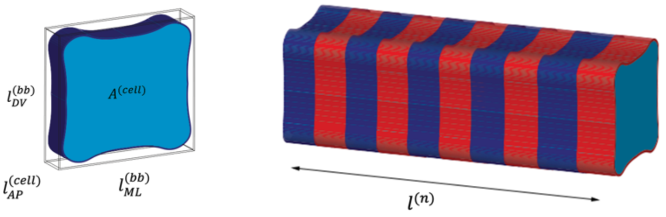
</td></tr>
<tr><td>Intercalation
$\beta(s)$ with transverse cross-sectional area
$A^{(cell)}(s)$</td><td>$n\,\beta(s)\frac{V^{(cell)}(s_0)}{A^{(cell)}(s)}$</td><td>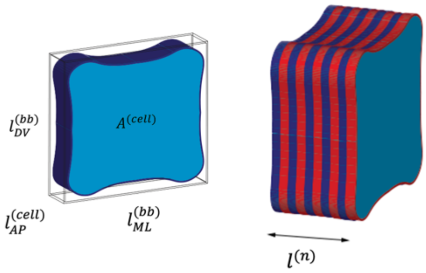
</td></tr>
<tr><td>Isotropic growth
$V^{(cell)}(s)$ without intercalation</td><td>$n\,\beta(s_0)\,l_{AP}^{(cell)}(s_0)\sqrt[3]{\frac{V^{(cell)}(s)}{V^{(cell)}(s_0)}}$</td><td>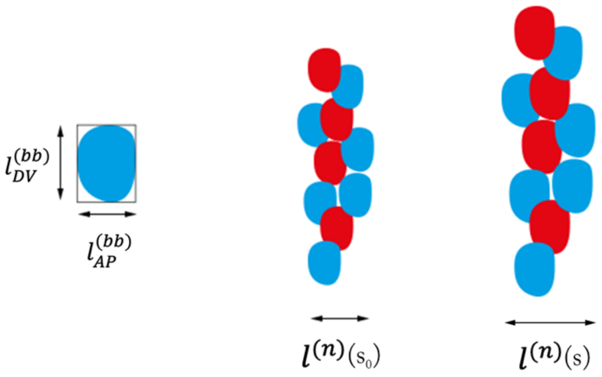
</td></tr>
<tr><td>Anisotropic growth
$V^{(cell)}(s)$ only along
AP axis $l_{AP}^{(cell)}(s)$,
without intercalation</td><td>$n\,\beta(s_0)\frac{V_{cell}(s)}{A^{(cell)}(s_0)}$</td><td>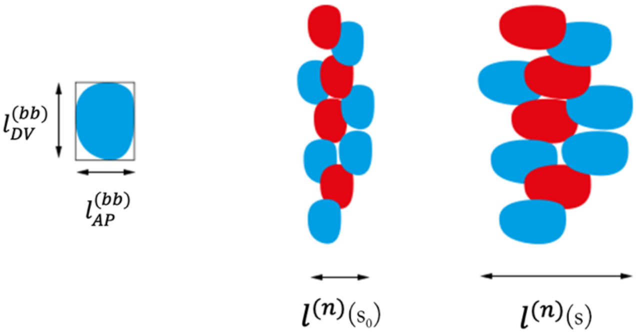
</td></tr>
</table>

Development • Supplementary information
